# Joint calibrated estimation of inverse probability of treatment and censoring weights for marginal structural models

**DOI:** 10.1111/biom.13411

**Published:** 2020-12-11

**Authors:** Sean Yiu, Li Su

**Affiliations:** ^1^ MRC Biostatistics Unit School of Clinical Medicine University of Cambridge Cambridge CB2 0SR UK

**Keywords:** calibration, causal inference, covariate balancing, dropout, longitudinal data, propensity score

## Abstract

Marginal structural models (MSMs) with inverse probability weighted estimators (IPWEs) are widely used to estimate causal effects of treatment sequences on longitudinal outcomes in the presence of time‐varying confounding and dependent censoring. However, IPWEs for MSMs can be inefficient and unstable if weights are estimated by maximum likelihood. To improve the performance of IPWEs, covariate balancing weight (CBW) methods have been proposed and recently extended to MSMs. However, existing CBW methods for MSMs are inflexible for practical use because they often do not handle dependent censoring, nonbinary treatments, and longitudinal outcomes (instead of eventual outcomes at a study end). In this paper, we propose a joint calibration approach to CBW estimation for MSMs that can accommodate (1) both time‐varying confounding and dependent censoring, (2) binary and nonbinary treatments, (3) eventual outcomes and longitudinal outcomes. We develop novel calibration restrictions by *jointly* eliminating covariate associations with both treatment assignment and censoring processes after weighting the observed data sample (i.e., to optimize covariate balance in finite samples). Two different methods are proposed to implement the calibration. Simulations show that IPWEs with calibrated weights perform better than IPWEs with weights from maximum likelihood and the “Covariate Balancing Propensity Score” method. We apply our method to a natural history study of HIV for estimating the effects of highly active antiretroviral therapy on CD4 cell counts over time.

## 1 INTRODUCTION

### 1.1 Marginal Structural Models and Covariate Balancing Weights

Marginal structural models (MSMs) (Robins, 1999b; Robins *et al*., 2000) with inverse probability of treatment weighting (IPTW) are widely used to estimate causal effects of treatment sequences on a longitudinal outcome in the presence of time‐varying confounders that are affected by treatment history (i.e., time‐varying confounding, Hernán *et al*., 2001; Daniel *et al*., 2013). With dependent censoring (e.g., due to loss of follow‐up of patients), MSMs are estimated by IPTW and inverse probability of censoring weighting (IPCW) that addresses the additional selection bias from censoring (Hernán *et al*., 2001).

To implement IPTW and IPCW for MSMs, time‐varying weights are commonly estimated by fitting parametric models for treatment assignment and censoring processes and then plugging in parameter estimates from maximum likelihood estimation (MLE). However, the MLE approach to weight estimation can result in inefficient and unstable inverse probability weighted estimators (IPWEs), especially when the treatment assignment and/or censoring model is misspecified (Kang and Schafer, 2007; Cole and Hernán, 2008; Lefebvre *et al*., 2008; Howe *et al*., 2011). Because final weights for fitting MSMs are a product of the time‐varying weights for IPTW and IPCW, the efficiency and stability issues of IPWEs can be exacerbated when both time‐varying confounding and dependent censoring are present.

Motivated by improving IPWEs primarily for binary point treatments, covariate balancing weight (CBW) methods, which directly optimize covariate balance for weight estimation, have been proposed (Graham *et al*., 2012; Hainmueller, 2012; Imai and Ratkovic, 2014; Zubizarreta, 2015; Chan *et al*., 2016; Yiu and Su, 2018; Fong *et al*., 2018). In empirical studies, CBW methods have been shown to dramatically improve the performance of IPWEs by reducing their mean squared errors (MSEs) under both correct and incorrect model specification. Recent theoretical investigations by Tan (2020) also reveal that, unlike the MLE approach, CBW methods can bound the MSEs of IPWEs even under model misspecification.

Recently, CBW methods have been extended to improve the IPWEs in MSMs. Imai and Ratkovic (2015) first extended the “Covariate Balancing Propensity Score” (CBPS) method to MSMs with binary treatments. However, because the number of moment conditions for weight estimation increases exponentially with the number of follow‐up visits, CBPS could only practically accommodate a small number of visits and covariates, and is computationally intensive. Yiu and Su (2018) demonstrated that their CBW framework for a general point treatment can be extended to estimate the *short‐term direct* effect of a time‐varying treatment on a longitudinal outcome. Nonetheless, it is often of greater interest to estimate the *total* effect of a treatment sequence in MSMs (i.e., both the direct treatment effect and the indirect treatment effect through time‐varying confounders on the longitudinal outcome). Focusing on MSMs with continuous treatments, Zhou and Wodtke (2020) proposed a different approach called “residual balancing”, where conditional means of time‐varying confounders are modeled, and weights for IPTW are estimated by balancing the residuals of the time‐varying confounder models across future treatments as well as the history of treatments and confounders. In practice, it is undesirable to model a whole set of time‐varying confounders and to specify the functional form of future treatments for balancing. A notable limitation of the CBW methods in Imai and Ratkovic (2015), Yiu and Su (2018), and Zhou and Wodtke (2020) is that they do not address the common problem of dependent censoring in MSMs. Kallus and Santacatterina (2019) developed “Kernel Optimal Weighting” to handle both time‐varying confounding and dependent censoring in MSMs. However, their approach is restricted to binary treatments and requires modeling conditional means of potential outcomes given observed histories of treatments and confounders. With the exception of Yiu and Su (2018), the aforementioned methods focused on MSMs for an *eventual* outcome at a study end, instead of MSMs for a longitudinal outcome over time, which are often of interest in practice. In summary, more research is required to improve the flexibility and practicality of CBW methods for their widespread use in longitudinal settings.

### 1.2 Joint Calibration Approach to Weight Estimation

To enhance the flexibility of CBW methods for practical use in MSMs, we propose a new calibration approach to CBW estimation that can accommodate (1) both time‐varying confounding and dependent censoring, (2) binary and nonbinary treatment sequences, and (3) eventual and longitudinal outcomes. Specifically, by building on the “Covariate Association Eliminating Weights” framework by Yiu and Su (2018) for point treatments, we propose novel moment conditions (i.e., calibration restrictions) for weight estimation that *jointly* remove covariate associations over time with both treatment assignment and censoring processes after weighting the observed data *sample* (i.e., to optimize covariate balance for both treatment assignment and censoring processes in finite samples).

The joint calibration of CBWs based on our proposed moment conditions can be implemented by three types of methods, as pointed out by a referee. These include: Type (1), calibrating an initial set of estimated weights (e.g., from MLE) with an exponential tilting term containing a parameter vector with dimension equal to the number of moment conditions (see Han (2016) for an example in handling dependent censoring); Type (2), estimating model‐based parameters of inverse probability weights by solving estimating equations based on the proposed moment conditions (e.g., Imai and Ratkovic, 2015); Type (3), estimating weights nonparametrically by solving a constrained optimization problem that incorporates the proposed moment conditions in the constraints (e.g., Zubizarreta, 2015; Chan *et al*., 2016; Yiu and Su, 2018; Kallus and Santacatterina, 2019; Zhou and Wodtke, 2020). We discuss the pros and cons of all three methods in the MSMs settings and develop both Types (1) and (2) methods for implementing the joint calibration in Section 4.3.

Besides flexibility, an important feature of our approach is its computational efficiency. This is because, (I) unlike the methods in Zhou and Wodtke (2020) and Kallus and Santacatterina (2019), it does not require models for the conditional means of time‐varying confounders or potential outcomes, (II) unlike the CBPS method in Imai and Ratkovic (2015), it allows for parsimony in deriving moment conditions when there exist many time‐varying confounders and visits, therefore the number of proposed moment conditions does not have to increase exponentially with the number of visits. For example, the number of moment conditions can increase linearly even if separate treatment assignment models are specified at each visit. Together the flexibility and computational efficiency of our approach can encourage more widespread and practical use of MSMs in complex longitudinal settings. Further details of the related literature and our contributions are provided in Web Appendix A.

As we focus on the main idea of the proposed calibration approach to CBWs, its implementation, and empirical evaluation in this paper, we leave the theoretical investigation of the robustness and efficiency of the proposed IPWEs for future work. In Section 7, we briefly discuss the robustness and efficiency issues regarding the proposed IPWEs in light of the recent theoretical development in the CBW literature (Wang and Zubizarreta, 2020).

### 1.3 Motivating Example

This research is motivated by data from the HIV Epidemiology Research Study (HERS), a natural history study of 1310 women with, or at high risk of, HIV infection at four sites (Baltimore, Detroit, New York, Providence) from 1993 to 2000 (Ko *et al*., 2003). During the study, 12 visits were scheduled, where a variety of clinical, behavioral, and sociological outcomes as well as self‐reported information on antiretroviral therapies (ARTs) were recorded approximately every 6 months.

Our objective is to quantify the effect of highly active antiretroviral therapy (HAART), which contains three or more ART regimens, on the CD4 cell counts over time in the HERS cohort. Because the HERS was an observational study, where therapies were not randomly assigned and varying over time, this leads to the potential for time‐varying confounding between treatment and outcome. Moreover, estimation of the treatment effect is further complicated by dependent censoring due to dropout: more than half of the 871 HIV‐infected women at enrollment did not complete the study. We provide further details about these problems in the HERS cohort in Web Appendix G.

In the previous analysis by Ko *et al*. (2003), weights for IPTW and IPCW were estimated by MLE to fit several MSMs and address the time‐varying confounding and dependent censoring problems in the HERS data. However, Ko *et al*. (2003) considered the time‐varying treatments to be binary (i.e., with and without HAART). Because patients on ART other than HAART (i.e., less than 3 ARTs) were combined with patients not receiving any treatment, the therapeutic effect of HAART relative to no treatment was likely to be underestimated. In this paper, we consider the time‐varying treatment as ordinal with 3 levels—“no treatment,” “ART other than HAART,” and “HAART,” which therefore allows more precise quantification of the effect of HAART. However, the probability of each level of the ordinal treatment can depend on many baseline and time‐varying covariates and their interactions (as reflected in the treatment guidelines when the HERS was conducted), which not only makes model misspecification likely but also makes it more challenging to balance the covariates across treatment levels. These issues thus motivated us to develop a new calibration approach to CBW estimation in MSMs.

## 2 NOTATION, SETTING AND ASSUMPTIONS

We consider a study in which *n* independent patients are enrolled at baseline (denoted by visit 0) and then followed up over time at visits j=1,…,T. For the *i*th patient, baseline covariates $V_{i}$ (e.g., demographics) are recorded. At each follow‐up visit *j*, we assume that this patient's treatment assignment $A_{ij}$, time‐varying covariate vector $X_{ij}$, and longitudinal outcome $Y_{ij}$ are measurable, and are recorded only if the patient makes the visit. We further assume that the variables follow the temporal ordering where $Y_{ij}$, $X_{ij}$, and $A_{ij}$ are only affected by $\left\{{\bar{A}}_{ij},{\bar{X}}_{ij},{\bar{Y}}_{i,j-1},V_{i}\right\}$, $\left\{{\bar{A}}_{ij},{\bar{X}}_{i,j-1},{\bar{Y}}_{i,j-1},V_{i}\right\}$, and $\left\{{\bar{A}}_{i,j-1},{\bar{X}}_{i,j-1},{\bar{Y}}_{i,j-1},V_{i}\right\}$, respectively, for j=1,…,T. Here an overbar is used to represent the history of a process, for example, ${\bar{X}}_{ij}=\left\{X_{i1},\text{…},X_{ij}\right\}$. For ease of exposition in what follows, we absorb ${\bar{Y}}_{i,j-1}$, ${\bar{A}}_{i,j-1}$ and $V_{i}$ into the covariate history ${\bar{X}}_{i,j-1}$ (j=1,…,T), unless stated otherwise.

Let $Y_{ij}^{{\bar{a}}_{j}}$ be the potential outcome that would have arisen at visit *j* had the *i*th patient been assigned the potential treatment sequence ${\bar{a}}_{j}$ from the first visit after baseline up to visit *j*. We assume that the causal effect of ${\bar{a}}_{j}$ on $Y_{ij}^{{\bar{a}}_{j}}$ can be encoded in an MSM of the form $E\left(Y_{ij}^{{\bar{a}}_{j}}\right)=\mu \left({\bar{a}}_{j},\gamma \right)=g\left\{h\left({\bar{a}}_{j}\right),\gamma \right\}$, where h(·) is a function satisfying $h({\bar{a}}_{j}=0)=0$, 0 is the vector of zeros, ${\bar{a}}_{j}=0$ is the potential treatment sequence where no treatment is administered at every visit up to visit *j*, and g(·) is a function that relates the mean of the potential outcome to $h({\bar{a}}_{j})$ through a finite‐dimensional parameter vector γ. Note that baseline covariates $V_{i}$ can also be included in the MSM. To identify and estimate γ from the observed data, we make the stable unit treatment value (SUTVA) assumption, that is, the distribution of potential outcomes for one patient is assumed to be independent of potential treatment sequence of another patient, and the potential outcomes are well defined. Additionally, we make the sequential ignorability of treatment assignment assumption, that is, $\text{pr}\left(A_{ij}|Y_{ij}^{{\bar{a}}_{j}},{\bar{X}}_{i,j-1}\right)=\text{pr}\left(A_{ij}|{\bar{X}}_{i,j-1}\right)$ for j=1,…,T, and the positivity assumption, that is, $\text{pr}(A_{ij}\in A|{\bar{X}}_{i,j-1})>0$ for all ${\bar{X}}_{i,j-1}$ and for any set A with positive measure. Note that $A_{ij}$ can have arbitrary distributions (e.g., ordinal and continuous).

In the presence of dependent censoring, the objective is to estimate the causal effect of the treatment sequence in MSMs without censoring, therefore further assumptions about the censoring process need to be made. Let $R_{ij}$ be the indicator of whether the *i*th patient remains in the study up to visit *j*. We assume that $R_{i0}=1$ (i.e., baseline visit assessments are complete for all patients) and $R_{i,j-1}=0\Rightarrow R_{ij}=0$ (monotone missingness due to dropout). Our interest is to estimate the parameters of the MSM for $E(Y_{ij}^{{\bar{a}}_{j},{\bar{r}}_{j}=1})$, where ${\bar{r}}_{j}$ is the potential sequence of the indicator of the *i*th patient being in the study by visit *j* and 1 is the vector of ones. To achieve this, we make an assumption that the censoring is sequentially ignorable, that is, $\text{pr}\left(R_{ij}|Y_{ij}^{{\bar{a}}_{j}},{\bar{H}}_{i,j-1},R_{i,j-1}=1\right)=\text{pr}\left(R_{ij}|{\bar{H}}_{i,j-1},R_{i,j-1}=1\right)$ for j=1,…,T, where ${\bar{H}}_{i,j-1}$ denotes the observable history of the *i*th patient up to visit j−1 that can include ${\bar{X}}_{i,j-1}$ and any other relevant covariate information. In addition, we assume that $\text{pr}(R_{ij}|{\bar{H}}_{i,j-1},R_{i,j-1}=1)>0$ for all ${\bar{H}}_{i,j-1}$, which is similar to the positivity assumption made for the treatment process.

Throughout the paper, we make the above assumptions; otherwise our method may result in severely biased estimates for parameters in the MSM, possibly even compared to an analysis without addressing time‐varying confounding and dependent censoring.

## 3 INVERSE PROBABILITY OF TREATMENT AND CENSORING WEIGHTING FOR MSMs

To identify and consistently estimate γ using observed data under the assumptions described in Section 2, the following inverse probability of treatment and censoring weighted (IPTCW) estimating equations

(1) $$\sum_{i=1}^{n}\sum_{j=1}^{T}R_{ij}SW_{ij}^{A}W_{ij}^{C}D\left({\bar{A}}_{ij},\gamma \right)\left\{Y_{ij}-\mu ({\bar{A}}_{ij},\gamma )\right\}=0$$

can be solved, where $SW_{ij}^{A}=\prod_{k=1}^{j}\text{pr}\left(A_{ik}|{\bar{A}}_{i,k-1}\right)/\prod_{k=1}^{j}\text{pr}\left(A_{ik}|{\bar{X}}_{i,k-1}\right)$ are the stabilized inverse probability of treatment weights, $W_{ij}^{C}=\prod_{k=1}^{j}1/\text{pr}(R_{ik}=1|{\bar{H}}_{i,k-1}$, $R_{i,k-1}=1)$ are the inverse probability of censoring weights, $D\left({\bar{A}}_{ij},\gamma \right)=\left\{\partial \mu \left({\bar{A}}_{ij},\gamma \right)/\partial \gamma \right\}V_{ij}^{-1}$, and $V_{ij}=\text{var}\left(Y_{ij}\right)$ (e.g., see Robins (1999b); Hernán *et al*. (2001); Ko *et al*. (2003) for proof). Other commonly used versions of (1) include replacing $W_{ij}^{C}$ with the stabilized weights for censoring, $SW_{ij}^{C}=W_{ij}^{C}\prod_{k=1}^{j}\text{pr}\left(R_{ik}=1|{\bar{A}}_{i,k-1},R_{i,k-1}=1\right)$, and incorporating baseline covariates $V_{i}$ in the numerator of $SW_{ij}^{A}$ (and $SW_{ij}^{C}$) when they are included in the MSM. For simplicity, we do not consider these alternatives here, but our proposed method described in Section 4 easily extends to these scenarios (e.g., see Web Appendix C for more details).

The purpose behind weighting the uncensored observation for the *i*th patient at visit *j* by $W_{ij}^{C}$ is to create a representative sample of the target population (in the absence of censoring) at visit *j*. This is achieved because the $\sum_{i=1}^{n}R_{ij}\left(W_{ij}^{C}-1\right)$ copies of the uncensored observations at visit *j* are representative of the censored observations up to and including visit *j* in terms of ${\bar{H}}_{i,j-1}$, and the remaining $\sum_{i=1}^{n}R_{ij}$ copies of the uncensored observations represent themselves (in total there are $\sum_{i=1}^{n}R_{ij}W_{ij}^{C}$ copies). That is, if * denotes the pseudo‐population after weighting the uncensored observations at visit *j* by $W_{ij}^{C}-1$, it can be shown that ${\text{pr}}^{*}\left(R_{ij}=1|{\bar{H}}_{i,j-1},R_{i0}=1\right)=1/2$ (see Web Appendix C for proof). Subsequently, the purpose of weighting the uncensored observations further by $SW_{ij}^{A}$ is to create a pseudo‐population where $A_{ij}$ is conditionally independent of ${\bar{X}}_{i,j-1}$ given ${\bar{A}}_{i,j-1}$, and the causal effect of ${\bar{a}}_{j}$ on $E(Y_{ij}^{{\bar{a}}_{j}})$ is the same as in the original population. Under the sequential ignorability, positivity, and SUTVA assumptions described in Section 2, the treatment process up to visit *j* after weighting by $SW_{ij}^{A}$ will then be causally exogenous (Robins, 1999b), that is, ${\text{pr}}^{*}\left(A_{ij}|Y_{ij}^{{\bar{a}}_{j}},{\bar{X}}_{i,j-1}\right)={\text{pr}}^{*}\left(A_{ij}|{\bar{X}}_{i,j-1}\right)=\text{pr}\left(A_{ij}|{\bar{A}}_{i,j-1}\right)$, where * denotes the pseudo‐population after weighting by $SW_{ij}^{A}W_{ij}^{C}$. Then standard regression methods can be used to consistently estimate γ in the MSM if the weights in (1) are known.

Because the weights in (1) are unknown in observational studies, their estimates based on MLE, $SW_{ij}^{A}\left(\hat{\alpha },\hat{\beta }\right)=\prod_{k=1}^{j}\text{pr}\left(A_{ik}|{\bar{A}}_{i,k-1};\hat{\alpha }\right)/\prod_{k=1}^{j}\text{pr}\left(A_{ik}|{\bar{X}}_{i,k-1};\hat{\beta }\right)$ and $W_{ij}^{C}\left(\hat{\theta }\right)=\prod_{k=1}^{j}1/\text{pr}\left(R_{ik}=1|{\bar{H}}_{i,k-1},R_{i,k-1}=1;\hat{\theta }\right)$ are usually used to implement IPTCW, where $\hat{\alpha }$, $\hat{\beta }$, and $\hat{\theta }$ are the maximum likelihood estimates of α, β, and θ in parametric models $\text{pr}(A_{ik}|{\bar{A}}_{i,k-1};\alpha )$, $\text{pr}(A_{ik}|{\bar{X}}_{i,k-1};\beta )$, and $\text{pr}(R_{ik}=1|{\bar{H}}_{i,k-1},R_{i,k-1}=1;\theta )$, respectively. However, as discussed in Section 1.1, this MLE approach to weight estimation can be problematic, which motivated CBW methods as an alternative.

## 4 JOINT CALIBRATED WEIGHT ESTIMATION FOR MSMs

In this section, we describe our joint calibration approach to CBW estimation in MSMs. It is important to note that the goal for calibration is to improve IPWEs of MSM parameters, and not to improve estimation of the treatment and censoring processes.

Specifically, we propose to calibrate $SW_{ij}^{A}\left(\hat{\alpha },\hat{\beta }\right)W_{ij}^{C}\left(\hat{\theta }\right)$ by jointly imposing calibration restrictions (i.e., moment conditions) implying that, after weighting the observed data sample, at each study visit (I) treatment assignments are unassociated with the history of time‐varying covariates, and (II) we have a representative sample of the target population in the absence of censoring. For the simpler setting of IPTW, $SW_{ij}^{A}\left(\hat{\alpha },\hat{\beta }\right)$ can be calibrated by only imposing the restrictions for the treatment assignment process.

Let $W_{ij}^{AC★}\left(\lambda \right)$ be the calibrated weights with parameter vector λ. Note that we use ⋆ in superscript to highlight that the weights are calibrated.

After obtaining an estimate of λ, $\hat{\lambda }$, we propose to replace $SW_{ij}^{A}W_{ij}^{C}$ by $W_{ij}^{AC★}\left(\hat{\lambda }\right)$ in (1) to estimate γ.

In the next sections, we derive calibration restrictions for the treatment assignment and censoring processes before developing the implementation procedures for the joint calibration.

### 4.1 Calibration Restrictions for Treatment Assignment

We derive calibration restrictions for treatment assignment by building on the framework proposed in Yiu and Su (2018) for point treatments. Let $\text{pr}(A_{ij}|{\bar{X}}_{i,j-1};\beta_{w})$ be a parametric model for the treatment assignment. Here we use the subscript “w” in $\beta_{w}$ to highlight that this is the parametric model used to derive restrictions. Following Yiu and Su (2018), we use the partition $\beta_{w}=\left\{\beta_{\text{wb}},\beta_{\text{wd}}\right\}$, where $\beta_{\text{wd}}$ are the unique parameters that characterize the dependence of $A_{ij}$ on ${\bar{X}}_{i,j-1}$ excluding the treatment history ${\bar{A}}_{i,j-1}$ (i.e., regression coefficients of baseline and time‐varying covariates and their interactions with ${\bar{A}}_{i,j-1}$), and $\beta_{\text{wb}}$ include the intercept terms and parameters that characterize the dependence on treatment history (i.e., regression coefficients of ${\bar{A}}_{i,j-1}$). Here the subscripts “d” and “b” stand for dependence and baseline, respectively. Without loss of generality, let $\text{pr}\left(A_{ij}|{\bar{X}}_{i,j-1};\beta_{\text{wb}}=\alpha ,\beta_{\text{wd}}=0\right)=\text{pr}\left(A_{ij}|{\bar{A}}_{i,j-1};\alpha \right)$, that is, setting $\left\{\beta_{\text{wb}}=\alpha ,\beta_{\text{wd}}=0\right\}$ results in a treatment process model that only depends on treatment history and is parameterized by α.

Now suppose that λ is fixed and we have *known* weights $W_{ij}^{AC★}\left(\lambda \right)$, it is possible to examine whether $\beta_{\text{wd}}=0$ in the weighted sample with $W_{ij}^{AC★}\left(\lambda \right)$, by finding the value of $\beta_{w}$, that is, ${\hat{\beta }}_{w}$, that maximizes

(2) $$\prod_{j=1}^{T}\prod_{i=1}^{n}{\left\{\prod_{k=1}^{j}\text{pr}\left(A_{ik}|{\bar{X}}_{i,k-1};\beta_{w}\right)\right\}}^{R_{ij}W_{ij}^{AC★}\left(\lambda \right)},$$

or equivalently solves the score equations

(3) $$\begin{array}{c} \sum_{j=1}^{T}\sum_{i=1}^{n}R_{ij}W_{ij}^{AC★}\left(\lambda \right)\sum_{k=1}^{j}\frac{\partial }{\partial \beta_{w}}\log\left\{\text{pr}\left(A_{ik}|{\bar{X}}_{i,k-1};\beta_{w}\right)\right\}=0. \end{array}$$

The terms in the curly brackets in (2) make it explicit that $W_{ij}^{AC★}\left(\lambda \right)$ is used to weight the likelihood of the observed treatment sequence for the *i*th patient up to visit *j*.

We propose to derive calibration restrictions by inverting (3) and finding the value of λ implying that $\left\{{\hat{\beta }}_{\text{wb}}=\hat{\alpha },{\hat{\beta }}_{\text{wd}}=0\right\}$ are the values that maximize (2). That is, we solve for λ

(4) $$\begin{array}{ccc} & & \sum_{j=1}^{T}\sum_{i=1}^{n}R_{ij}W_{ij}^{AC★}\left(\lambda \right)\sum_{k=1}^{j}\frac{\partial }{\partial \beta_{w}}\log\left\{\text{pr}\left(A_{ik}|{\bar{X}}_{i,k-1};\beta_{w}\right)\right\}\\ & & {\;|}_{\left\{\beta_{\text{wb}}=\hat{\alpha },\beta_{\text{wd}}=0\right\}}=0. \end{array}$$

Satisfaction of the restrictions in (4) means that, after weighting by $W_{ij}^{AC★}\left(\hat{\lambda }\right)$, the treatment assignments up to visit *j* are unassociated with the covariate histories conditional on the treatment histories in the observed data sample (i.e., ${\hat{\beta }}_{\text{wd}}=0$). Note that the structure of the covariate associations is characterized by the specified parametric treatment process model. More discussion about this general framework for weight estimation for point treatments can be found in Yiu and Su (2018). In addition, Web Appendix B provides details of deriving calibration restrictions for the eventual outcome setting.

#### 4.1.1 Application to ordinal treatments

We consider the following model for the ordinal treatment variable in the HERS data,

(5) $$\begin{array}{cc} & \mathrm{logit}\left\{\text{pr}\left(A_{ij}^{0}=1|{\bar{X}}_{i,j-1}\right)\right\}={\tilde{X}}_{i,j-1}^{0⊤}\beta^{0},\\ & \mathrm{logit}\left\{\text{pr}\left(A_{ij}^{1}=1|{\bar{X}}_{i,j-1},A_{ij}^{0}=1\right)\right\}={\tilde{X}}_{i,j-1}^{1⊤}\beta^{1}, \end{array}$$

where $A_{ij}^{0}$ is the indicator of whether at least one ART was administered, $A_{ij}^{1}$ is the indicator of whether HAART was administered, ${\tilde{X}}_{i,j-1}^{0}$ and ${\tilde{X}}_{i,j-1}^{1}$ are functionals of ${\bar{X}}_{i,j-1}$ (e.g., transformations and interactions) including 1, and $\beta^{0}$ and $\beta^{1}$ are corresponding regression coefficients. Applying (4), restrictions based on (5) can be derived as

(6) $$\begin{array}{cc} \sum_{j=1}^{T}\sum_{i=1}^{n}R_{ij}W_{ij}^{AC★}\left(\lambda \right)\sum_{k=1}^{j}\left(A_{ik}^{0}-{\hat{e}}_{ik}^{0}\right){\tilde{X}}_{i,k-1}^{0} & =0,\\ \sum_{j=1}^{T}\sum_{i=1}^{n}R_{ij}W_{ij}^{AC★}\left(\lambda \right)\sum_{k=1}^{j}A_{ik}^{0}\left(A_{ik}^{1}-{\hat{e}}_{ik}^{1}\right){\tilde{X}}_{i,k-1}^{1} & =0, \end{array}$$

where ${\hat{e}}_{ik}^{0}$ and ${\hat{e}}_{ik}^{1}$ are the predicted probabilities of receiving treatment at visit *k* from fitting the model (5) but with treatment history as the only covariates. The restrictions in (6) are in spirit similar to the covariate balancing restrictions/conditions for binary point treatments (Imai and Ratkovic, 2014; Yiu and Su, 2018), but they are aggregated over time. Examining these restrictions carefully, we can see that they aim to remove the associations of the covariates, ${\tilde{X}}_{i,j-1}^{0}$ and ${\tilde{X}}_{i,j-1}^{1}$, with the residuals of the treatment variables (after fitting (5) with treatment history as the only covariates) over time.

#### 4.1.2 Application to continuous treatments

We assume that a time‐varying continuous treatment at visit *j* for the *i*th patient follows a heteroscedastic normal linear model $A_{ij}∼N\left\{{\tilde{X}}_{i,j-1}^{\mu ⊤}\beta^{\mu },\exp\left({\tilde{X}}_{i,j-1}^{\sigma ⊤}\beta^{\sigma }\right)\right\}$, where ${\tilde{X}}_{i,j-1}^{\mu }$ and ${\tilde{X}}_{i,j-1}^{\sigma }$ include 1 and functionals of ${\bar{X}}_{i,j-1}$ (e.g., interactions), and $\beta^{\mu }$ and $\beta^{\sigma }$ are corresponding regression coefficients. After applying (4) to this model for treatment assignment, we obtain the following restrictions

$$\begin{array}{c} \sum_{j=1}^{T}\sum_{i=1}^{n}R_{iT}W_{ij}^{AC★}\left(\lambda \right)\sum_{k=1}^{j}\frac{\left(A_{ik}-{\hat{\mu }}_{ik}\right)}{{\hat{\sigma }}_{ik}^{2}}{\tilde{X}}_{i,k-1}^{\mu }=0,\\ \sum_{j=1}^{T}\sum_{i=1}^{n}R_{iT}W_{ij}^{AC★}\left(\lambda \right)\sum_{k=1}^{j}\left\{-1+\frac{{\left(A_{ik}-{\hat{\mu }}_{ik}\right)}^{2}}{{\hat{\sigma }}_{ik}^{2}}\right\}{\tilde{X}}_{i,k-1}^{\sigma }=0, \end{array}$$

where ${\hat{\mu }}_{ik}$ and ${\hat{\sigma }}_{ik}^{2}$ are the estimated mean and variance of the continuous treatments from fitting the same normal linear model but with treatment history as the only covariates. It is easy to see that these restrictions are designed to remove the associations between the covariates ${\tilde{X}}_{i,k-1}^{\mu }$, ${\tilde{X}}_{i,k-1}^{\sigma }$ and the standardized residuals of a treatment model that depends only on treatment history.

#### 4.1.3 Additional restrictions for IPTW when implementing with the Type (1) method

When the Type (1) method is used to implement IPTW only (e.g., when censoring is assumed to depend on treatment history only), we propose to estimate calibrated weights $W_{ij}^{A★}\left(\lambda \right)$ for IPTW (instead of $W_{ij}^{AC★}\left(\lambda \right)$ for IPTCW) by jointly imposing (4) and additional restrictions

(7) $$\sum_{i=1}^{n}R_{ij}W_{ij}^{A★}\left(\lambda \right)=\sum_{i=1}^{n}R_{ij}$$

for j=1,…,T, in the same spirit as in Cao *et al*. (2009). That is, we also constrain the average of the calibrated weights to equal 1 at each visit. The purpose of these restrictions is to avoid the trivial solution of zeros for the weights in (4), and to improve the stability of the IPWE by prohibiting extremely large weights. As we shall see in Section 4.2, the calibration restrictions for IPCW already impose constraints on the sample size after weighting. Therefore, restrictions in (7) are redundant when calibrating weights for IPTCW.

### 4.2 Calibration Restrictions for Censoring

To derive calibration restrictions for IPCW, we utilize the proposition that at visit *j* (j=1,…,T), IPCW creates a representative sample of the target population (in the absence of censoring) at visit *j* after weighting the uncensored observations by $W_{ij}^{C}$. This proposition can be proved by induction (see Web Appendix C). An important step in the proof is to validate the inductive steps up to visit *j*, that is, weighting the uncensored observations at visit k=1,…,j by $1/\pi_{ik}-1$, where $\pi_{ik}=\text{pr}\left(R_{ik}=1|{\bar{H}}_{i,k-1},R_{i,k-1}=1\right)$, creates a representative sample of the *censored* observations at visit *k*, assuming that the proposition holds at visit k−1 and the uncensored observations at visit k−1 have been weighted by $W_{i,k-1}^{C}$. Therefore we derive calibration restrictions for censoring by inverting weighted score equations of a parametric model evaluated at the point implying *no evidence against* the inductive steps.

Specifically, suppose that λ is fixed and the calibrated weights $W_{i1}^{AC★}\left(\lambda \right),\text{…},W_{ij}^{AC★}\left(\lambda \right)$ are known. We can assess the validity of the proposition at visit *j* by specifying a parametric model $\pi_{ik}\left(\theta_{w}\right)=\text{pr}\left(R_{ik}=1|{\bar{H}}_{i,k-1},R_{i,k-1}=1;\theta_{w}\right)$ (k=1,…,j) and estimating its parameter $\theta_{w}$ by maximizing

(8) $$\begin{array}{ccc} & & L_{j}\left(\theta_{w}\right)=\prod_{i=1}^{n}\prod_{k=1}^{j}\\ & & \;{\left[\pi_{ik}{\left(\theta_{w}\right)}^{R_{ik}\left\{1/\pi_{ik}^{★}\left(\lambda \right)-1\right\}}{\left\{1-\pi_{ik}\left(\theta_{w}\right)\right\}}^{1-R_{ik}}\right]}^{W_{i,k-1}^{AC★}\left(\lambda \right)R_{i,k-1}}, \end{array}$$

where $1/\pi_{ik}^{★}\left(\lambda \right)=W_{ik}^{AC★}\left(\lambda \right)/W_{i,k-1}^{AC★}\left(\lambda \right)$ (k=1,…,j), $W_{i0}^{AC★}\left(\lambda \right)=1$, and by convention 0^0^ = 1. The terms in (8) are used to assess the validity of the inductive steps at visits k=1,…,j in the observed data sample, given that weighting by $W_{i,k-1}^{AC★}$ has been applied at visit k−1. In particular, if $\pi_{ik}\left(\theta_{w}=0\right)=1/2$ ∀k, deviations from ${\hat{\theta }}_{w}=0$ in the observed data sample would provide evidence against the inductive step at one or more visits up to and including visit *j*, and thus evidence against the proposition at visit *j*. Similarly, we can simultaneously assess the validity of the proposition at all visits by maximizing

(9) $$\begin{array}{ccc} & & \prod_{j=1}^{T}L_{j}\left(\theta_{w}\right)=\prod_{j=1}^{T}\prod_{i=1}^{n}\\ & & \;{\left[\pi_{ij}{\left(\theta_{w}\right)}^{R_{ij}\left\{1/\pi_{ij}^{★}\left(\lambda \right)-1\right\}}{\left\{1-\pi_{ij}\left(\theta_{w}\right)\right\}}^{1-R_{ij}}\right]}^{\left(T-j+1\right)W_{i,j-1}^{AC★}\left(\lambda \right)R_{i,j-1}} \end{array}$$

with the score equations

(10) $$\begin{array}{ccc} & & \sum_{j=1}^{T}\sum_{i=1}^{n}\left(T-j+1\right)\left[R_{ij}\left\{W_{ij}^{AC★}\left(\lambda \right)-W_{i,j-1}^{AC★}\left(\lambda \right)\right\}\frac{\partial }{\partial \theta_{w}}\log\left\{\pi_{ij}\left(\theta_{w}\right)\right\}\right.\\ & & \;+\;\left.W_{i,j-1}^{AC★}\left(\lambda \right)\left(R_{i,j-1}-R_{ij}\right)\frac{\partial }{\partial \theta_{w}}\log\left\{1-\pi_{ij}\left(\theta_{w}\right)\right\}\right]=0. \end{array}$$

The terms in square brackets in (9) are weighted by T−j+1 because they are required for assessing whether the proposition holds at visits j,…,T. We derive restrictions by finding λ such that ${\hat{\theta }}_{w}=0$ are the values that solve (10). That is, we solve for λ such that

(11) $$\begin{array}{ccc} & & \sum_{j=1}^{T}\sum_{i=1}^{n}\left(T-j+1\right)\left[R_{ij}\left\{W_{ij}^{AC★}\left(\lambda \right)-W_{i,j-1}^{AC★}\left(\lambda \right)\right\}\frac{\partial }{\partial \theta_{w}}\log\left\{\pi_{ij}\left(\theta_{w}\right)\right\}\right.\\ & & \;+\;\left.W_{i,j-1}^{AC★}\left(\lambda \right)\left(R_{i,j-1}-R_{ij}\right)\frac{\partial }{\partial \theta_{w}}\log\left\{1-\pi_{ij}\left(\theta_{w}\right)\right\}\right]{|}_{\theta_{w}=0}=0. \end{array}$$

In this paper, we assume a logistic model $\mathrm{logit}\left\{\pi_{ij}\left(\theta_{w}\right)\right\}={\tilde{H}}_{i,j-1}^{⊤}\theta_{w}$, where ${\tilde{H}}_{i,j-1}$ is a vector of functionals of ${\bar{H}}_{i,j-1}$ including 1. Then the restrictions based on (11) are

(12) $$\begin{array}{ccc} & & \sum_{j=1}^{T}\left(T-j+1\right)\sum_{i=1}^{n}\left[R_{ij}W_{ij}^{AC★}\left(\lambda \right)-R_{i,j-1}W_{i,j-1}^{AC★}\left(\lambda \right)\right]{\tilde{H}}_{i,j-1}=0. \end{array}$$

The term $\sum_{i=1}^{n}\left[R_{ij}W_{ij}^{AC★}\left(\lambda \right)-R_{i,j-1}W_{i,j-1}^{AC★}\left(\lambda \right)\right]{\tilde{H}}_{i,j-1}$ in (12) can be interpreted as the covariate balance summary of ${\tilde{H}}_{i,j-1}$ between the weighted uncensored observations at visit *j* and the weighted uncensored observations at visit j−1. Equation in (12) is equivalent to

(13) $$\begin{array}{c} \sum_{j=1}^{T}\sum_{i=1}^{n}R_{ij}W_{ij}^{AC★}\left(\lambda \right)\left\{\left(T-j+1\right){\tilde{H}}_{i,j-1}-\left(T-j\right){\tilde{H}}_{ij}\right\}=T\sum_{i=1}^{n}{\tilde{H}}_{i0} \end{array}$$

(see details in Web Appendix C). Since ${\tilde{H}}_{i,j-1}$ (j=1,…,T) includes 1, (13) implies $\sum_{j=1}^{T}\sum_{i=1}^{n}R_{ij}W_{ij}^{AC★}\left(\lambda \right)=nT$, which means that the total number of “observations” after weighting is equal to nT, the total number of observations of the target population without censoring. If ${\tilde{H}}_{i,j-1}$ includes baseline covariates $V_{i}$, (13) implies $\sum_{j=1}^{T}\sum_{i=1}^{n}R_{ij}W_{ij}^{AC★}\left(\lambda \right)V_{i}=T\sum_{i=1}^{n}V_{i}$, that is, the weighted average of $V_{i}$ over all visits is equal to the sample average of $V_{i}$. If ${\tilde{H}}_{i,j-1}$ includes an indicator for visit, I(j=k) (k=1,…,T), and an interaction between this visit indicator and $V_{i}$, $I\left(j=k\right)V_{i}$, then (13) implies $\sum_{i=1}^{n}R_{ik}W_{ik}^{AC★}\left(\lambda \right)=n$ and $\sum_{i=1}^{n}R_{ik}W_{ik}^{AC★}\left(\lambda \right)V_{i}=\sum_{i=1}^{n}V_{i}$ for k=1,…,T. That is, at each visit the sample size after weighting is *n* and the weighted average of $V_{i}$ is equal to the sample average of $V_{i}$. Note that interactions between visits and time‐varying covariates can also be included in ${\tilde{H}}_{i,j-1}$, therefore time‐varying covariates at different visits can be balanced separately.

In this section, we have derived restrictions for calibrating unstabilized weights for censoring; restrictions for stabilized weights for censoring can be found in Web Appendix C.

### 4.3 Implementation of the Joint Calibration

#### 4.3.1 Pros and cons of implementation methods

We discuss the pros and cons of the three types of implementation methods for calibration introduced in Section 1.2. The advantage of Type (1) methods is that they almost always result in a unique solution and they are computationally efficient. This is explained in Section 4.3.3 by showing that Type (1) methods are equivalent to solving a convex minimization problem. The disadvantage of Type (1) methods is that they can inherit the poor performance of the initial weights, for example, when the initial weights are generated by a severely misspecified model. Type (2) methods do not have this problem because no initial weights are required. However, they are not guaranteed to produce weights that satisfy the calibration restrictions exactly, not to mention a unique set of weights if such a set exists. This problem is especially prominent for long treatment sequences and complex models involving nonbinary treatments. For example, in our first simulation study in Section 5, the Type (2) method in Section 4.3.4 worked well when only IPTW was required, but failed to converge (i.e., did not find weights that satisfied our proposed moment conditions) for the majority of simulated data sets when IPTCW was applied. Furthermore, in our HERS data example, the same Type (2) method produced several sets of weights that differed by more than a constant of proportionality, that is, there existed multiple solutions.

Type (3) methods look promising because they seem not to suffer from the disadvantages of Type (1) and (2) methods. Nevertheless, we discourage using Type (3) methods to implement our calibration approach because (a) they may not result in a consistent estimator of treatment effects in MSMs even when the models for deriving calibration restrictions are correctly specified, and (b) they can be computationally intensive. Issue (a) arises because in longitudinal settings, unlike Type (1) and (2) methods, Type (3) methods do not impose enough structure on the weights to ensure that they converge to the true weights for IPTCW. One way to address issue (a) is to impose more calibration restrictions by either specifying and/or modeling conditional means of the time‐varying confounders given observed histories (Zhou and Wodtke, 2020), or conditional means of the potential outcomes given observed histories (Kallus and Santacatterina, 2019). However, in practice, it would be cumbersome and undesirable to conduct additional complex modeling, apart from specifying the treatment and censoring models for IPTCW in MSMs.

#### 4.3.2 Generic estimation procedure for joint calibration

For ease of exposition, we collect all standard IPTCW weights by MLE $SW_{ij}^{A}\left(\hat{\alpha },\hat{\beta }\right)W_{ij}^{C}\left(\hat{\theta }\right)$ and calibrated weights $W_{ij}^{AC★}\left(\lambda \right)$ into two m×1 vectors $W(\hat{\alpha },\hat{\beta },\hat{\theta })$ and $W^{★}\left(\lambda \right)$, respectively, where *m* is the number of weights. If no censoring occurs, then m=nT. Here, $\hat{\alpha }$, $\hat{\beta }$, and $\hat{\theta }$ denote parameter estimates by MLE *without weighting*. The implementation of joint calibration requires solving a system of linear equations in terms of $W^{★}\left(\lambda \right)$ since the restrictions (4), (7), and (13) are linear in the calibrated weights. Let K be the *known* m×r matrix and l be the *known* r×1 vector, where *r* is the numbers of restrictions. For example, for IPTCW, *r* would be the combined size of $\beta_{w}$ and $\theta_{w}$. Both K and l are determined by the calibration restrictions (4), (7), and (13). For obtaining the calibrated weights, we need to solve

(14) $$K^{⊤}W^{★}\left(\lambda \right)-l=0,$$

which can be performed in R (R Development Core Team, 2014) by using the package nleqslv (Hasselman, 2018), once the form of the calibrated weights $W^{★}\left(\lambda \right)$ has been specified. The forms of the calibrated weights in the Type (1) and (2) methods differ and are now specified in the following sections.

#### 4.3.3 Calibrated weights in the Type (1) method

We consider calibrated weights of the form $W^{★}\left(\lambda \right)=W\left(\hat{\alpha },\hat{\beta },\hat{\theta }\right)\circ \exp\left(K\lambda \right)$ for the Type (1) method, where exp(·) is performed element‐wise, ○ denotes element‐wise product, and λ is an r×1 vector of parameters. Although other forms of calibration are possible (e.g., see Han (2016)), this particular choice is appealing because solving (14) is equivalent to minimizing the convex function for λ,

(15) $$1^{⊤}\left\{W\left(\hat{\alpha },\hat{\beta },\hat{\theta }\right)\circ \exp\left(K\lambda \right)\right\}-l^{⊤}\lambda ,$$

where 1 is an m×1 vector of ones. The convexity of (15) ensures that the solution to (14) is unique and can be found efficiently. For the HERS analysis in Section 6, it took approximately two seconds to obtain the calibrated weights by imposing 84 restrictions for 2581 observations on a Linux machine with 2.40GHz CPU (four processors) and 128 GB memory.

#### 4.3.4 Calibrated weights in the Type (2) method

For the Type (2) method, we consider calibrated weights of the form $W^{★}\left(\lambda \right)=W\left(\hat{\alpha },\beta ,\theta \right)$. That is, the calibrated weights take the form of the standard IPTCW weights. However, the parameters characterizing these weights, λ={β,θ}, are estimated by solving  (14) once α has been estimated by MLE. Recall that if only IPTW is to be applied using the Type (2) method, (14) will only include restrictions (4) (not additional restrictions (7)) in order to ensure that the number of parameters to be estimated is equal to the number of moment conditions. Unlike the Type (1) method, this Type (2) method resulted in multiple solutions in the HERS data example, and failed to converge when IPTCW was applied in both the HERS data example and simulation studies.

#### 4.3.5 Other practical guidelines

It can be shown that the true inverse probability weights must satisfy the proposed moment conditions asymptotically, that is, the calibration/exponential tilting function in the Type (1) method should converge to 0 if the initial weights are estimated from a correctly specified model (see Web Appendix F for proof). Thus the proposed moment conditions can also be used for model checking. For example, in the HERS data analysis, we calculated the variance of the estimated calibration function since, if the treatment and censoring models for the initial weights are correctly specified, this variance should be close to zero (see Section 7.4 in Web Appendix G). Alternatively, one could detect whether a particular set of weights approximate the true IPTCW weights by assessing how close these weights are to satisfying the proposed moment conditions.

We distinguish between covariate histories that are predictive of $E(Y_{ij}^{{\bar{a}}_{j},{\bar{r}}_{j}=1})$, denoted as ${\bar{X}}_{i,j-1}^{Y}$, and those that are predictive of $A_{ij}$, denoted as ${\bar{X}}_{i,j-1}^{A}$. Some elements of ${\bar{X}}_{i,j-1}^{Y}$ and ${\bar{X}}_{i,j-1}^{A}$ overlap, which leads to confounding bias. We recommend prioritizing ${\tilde{X}}_{i,j-1}^{Y}$, that is, functionals of ${\bar{X}}_{i,j-1}^{Y}$, for inclusion in the models of the treatment and censoring processes for deriving restrictions at visit *j* (Zhao and Percival, 2017). In Web Appendix D, we provide further discussion on model choices for deriving calibration restrictions.

## 5 SIMULATION

We conduct two simulation studies to assess the finite sample performance of IPWEs for MSMs based on our calibration approach. The set‐up of the first simulation study is motivated by the HERS data, where ordinal time‐varying treatments are observed over long follow‐up periods and dependent censoring is present. Because the CBPS approach of Imai and Ratkovic (2015) can only handle binary treatments with a small number of visits, we are only able to compare the performance of our calibration approach with the MLE approach in the first simulation study. To include the CBPS approach for comparison, we design a second simulation study for binary treatments at five follow‐up visits and with no censoring. The computing time for the CBPS approach implemented by the CBPS package in R is 800 or more times than those for the calibration and MLE approaches. Full details of the simulation studies can be found in Web Appendix E. The R code for the simulation study is also available in the Supporting Information.

Overall, the simulation results confirm that both IPWEs for MSMs with weights from MLE and our calibration approach (implemented by both Type (1) and Type (2) methods) have negligible bias when the treatment and censoring models are correctly specified. IPWEs from the CBPS approach have small amounts of biases when the treatment model is correctly specified. However, with model misspecification for weight estimation, IPWEs from all approaches may have large biases that do not disappear with increasing sample sizes. Notably, the IPWEs with calibrated weights are considerably less variable and have much smaller MSEs than their MLE counterparts, especially when the treatment assignment and censoring models are misspecified. In particular, when model misspecification is induced by functional form misspecification of the covariates, the IPWEs with weights from MLE have large variances and MSEs that even increase with sample size. In contrast, the IPWEs with our calibrated weights are more stable and have smaller MSEs that decrease with sample size. The IPWEs with calibrated weights also have smaller median absolute errors (MAEs) and MSEs than their CBPS counterparts. The MAE, which is more robust to extreme values than the MSE, indicates that, after throwing away the worst half of the simulations results, our calibration approach still performs better than the CBPS approach.

## 6 APPLICATION

In this section, we apply the proposed method to the HERS data. Since HAART was not available at enrollment in the HERS cohort, we follow Ko *et al*. (2003) and treat visit 7, when HAART was more widely used in the HERS, as the “baseline” and estimate the causal effects of HAART over the 2‐year period between visit 8 and visit 12. Besides attrition, there were secondary sources of missing data that resulted in intermittent missing data (before being lost to follow‐up), missing data at enrollment for CD4 counts, and left‐censored HIV viral load at the lower detection limit. We deal with these by following the approaches in Ko *et al*. (2003); see Web Appendix G for details. In total, there are 610 patients at visit 7 who had at least one CD4 count measured between visit 8 and 12 and sufficient information for covariates to estimate the weights for IPTW and IPTCW. The total number of CD4 count observations for analysis is 2581.

### 6.1 Model Parameterizations and Estimation

As discussed in Section 1.3, in order to provide a more precise estimate of the causal effect of HAART relative to no treatment, we consider the time‐varying antiretroviral treatment assignment as an ordinal variable, which is represented by the indicator of whether at least one ART was administered, $A_{ij}^{0}$ (with a potential value $a_{j}^{0}$), and the indicator of whether HAART was administered, $A_{ij}^{1}$ (with a potential value $a_{j}^{1}$), for j=8,…,12. Let $D_{i}=0$ if $Y_{i7}<200$, $D_{i}=1$ if $200\leq Y_{i7}\leq 500$, and $D_{i}=2$ if $Y_{i7}>500$, where $Y_{i7}$ is the CD4 count at visit 7. We specify the following MSM for the potential CD4 count outcome $Y_{ij}^{{\bar{a}}_{j}}$,

$$\begin{array}{ccc} E(Y_{ij}^{{\bar{a}}_{j}}) & = & \delta_{0j}+\sum_{k=1}^{2}\delta_{k}I\left(D_{i}=k\right)+\delta_{v}^{⊤}V_{i}+\sum_{k=0}^{2}I\left(D_{i}=k\right)\\ & & \times \;\left\{\gamma_{1k}\sum_{l=8}^{j}\left(a_{l}^{0}-a_{l}^{1}\right)+\gamma_{2k}\sum_{l=8}^{j}a_{l}^{1}\right\} \end{array}$$

for j=8,…,12, where $\delta_{0j}$ are visit‐specific intercept terms, $V_{i}$ are baseline covariates evaluated at visit 7 (see the full list in Web Appendix G), and $\delta_{v}$ are their corresponding regression coefficients. This MSM encodes the cumulative effect of HAART and 1‐2 ARTs relative to no treatment stratified by the CD4 count at visit 7. If $\gamma_{1k}$ and $\gamma_{2k}$ are constrained to be constant across *k* (k=0,1,2 for the strata), then an overall cumulative treatment effect can be obtained. In addition, we assume a different MSM for evaluating short‐term treatment effects in Web Appendix G.

The parameters in the MSMs were estimated by applying IPTW and IPTCW, with weights estimated by MLE and our calibration approach with the Type (1) method. As mentioned previously, we consider the Type (2) method as an unreliable option for weight estimation in the HERS data because it produced multiple solutions for IPTW and did not converge for IPTCW. For IPTW, we assume the model in (5) for the MLE approach and for deriving restrictions for calibration.

In Web Appendix G, we provide the full list of covariates in (5).

For IPTCW, a logistic model with the same covariates as those in the treatment assignment model was used for estimating the inverse probability of censoring weights by MLE and for deriving calibration restrictions (12). To prevent extreme weights as in Cao *et al*. (2009), the weights in IPTW by the MLE approach were scaled to sum to the number of observations in the HERS data (i.e., 2581); and the weights from MLE for IPTCW were scaled to sum to 5 times the sample size at visit 7 (i.e., the number of outcome measurements that would have been observed had nobody been censored from visit 7 onward). Finally, we estimated standard errors with 2500 nonparametric bootstrap samples by treating patients as resampling units.

### 6.2 Results

In Web Appendix G, we provide details of the estimated weights and discuss the extent to which they suggest that confounding bias from observed covariates is present and the positivity assumption is satisfied.

Table 1 presents the estimates and standard errors of the parameters in the specified MSMs with no weighting, IPTW, and IPTCW. The results of the naïve analysis with no weighting applied, as shown in the first two rows of Table 1, strongly suggest that, compared with no treatment, HAART was effective at increasing the CD4 counts over time for those with CD4≤500 at visit 7, and 1‐2 ARTs were effective for those with 200≤CD4≤500 at visit 7. However, point estimates for the group with CD4>500 at visit 7 showed detrimental effects of both HAART and 1‐2 ARTs.

**TABLE 1:** Parameter estimates and bootstrap standard errors of the MSMs by applying no weighting, IPTW, and IPTCW with weights from maximum likelihood (MLE) and from the calibration approach (CMLE) to the HERS data

		Strata by CD4 cell count at visit 7			
Weight estimation	Cumulative effect	<200	200–500	>500	No stratification
	*No weighting*				
	⩽2 ARTs	8.57 (9.33)	13.66 (7.86)	−27.36 (18.40)	0.51 (8.06)
	HAART	26.34 (8.37)	27.40 (8.25)	−25.59 (16.45)	14.46 (7.99)
	*Treatment only*				
MLE	⩽2 ARTs	13.27 (9.84)	16.23 (8.71)	−26.44 (23.06)	5.59 (9.58)
	HAART	27.78 (9.69)	28.63 (10.24)	−2.67 (23.16)	20.89 (10.04)
CMLE	⩽2 ARTs	14.35 (9.09)	26.60 (7.60)	5.25 (18.09)	18.59 (7.23)
	HAART	36.53 (8.09)	34.73 (7.88)	−2.75 (17.87)	28.16 (7.36)
	*Treatment and dropout*				
MLE	⩽2 ARTs	11.70 (9.29)	17.11 (8.57)	−24.75 (22.74)	6.84 (9.07)
	HAART	25.79 (9.19)	28.80 (10.49)	−2.08 (22.39)	21.19 (9.72)
CMLE	⩽2 ARTs	11.92 (8.67)	27.74 (7.66)	8.60 (17.74)	19.26 (7.08)
	HAART	33.11 (7.93)	32.75 (8.00)	3.10 (16.94)	27.37 (7.24)

Applying IPTW with weights from MLE provides an upward adjustment of the treatment effects, as seen in the third and fourth rows of Table 1. The largest adjustments for 1‐2 ARTs and HAART are in the CD4<200 and CD4>500 strata, respectively. Overall, this results in a fairly substantial upward adjustment for the treatment effects in the MSM with no stratification. However, applying IPTW with weights from MLE also increased the standard errors of the estimated treatment effects.

The fifth and sixth rows in Table 1 present the results from applying IPTW with calibrated weights from the Type (1) method. It appears that HAART had an even greater effect on increasing CD4 counts for those with CD4≤500 at visit 7 and overall without stratification, compared with the results based on weights from MLE. There were also substantial increases in the estimated effects of 1‐2 ARTs for those with ≥200 and overall. As anticipated, the estimated standard errors with the calibrated weights are much smaller even compared to the naïve analysis with no weighting applied.

Further adjustment for selection bias due to dependent censoring appears to have largely minor effects, as seen in the last four rows of Table 1. The most notable modifications occur in the CD4>500 strata. However, there is substantial uncertainty associated with these estimated treatment effects, therefore the evidence is insufficient to draw a conclusion.

As expected, our estimated treatment effects for HAART are generally much larger (more than 1 standard error) than those reported in Ko *et al*. (2003), since we have separated the group with 1‐2 ARTs from the group with no treatment. The slightly larger effect of HAART in the CD4>500 strata from Ko *et al*. (2003) is again associated with substantial uncertainty.

In conclusion, the results in Table 1 indicate that there were clinically substantial and statistically significant therapeutic effects of cumulative exposure to HAART for those patients with initial CD4 count ⩽500, which is consistent with the findings in Ko *et al*. (2003) and the recommended treatment guideline during the study period of the HERS.

## 7 CONCLUSION AND DISCUSSION

In this paper, we have proposed a new CBW approach to MSMs that can accommodate both time‐varying confounding and dependent censoring, binary and nonbinary time‐varying treatments as well as eventual and longitudinal outcomes. Simulations showed that IPWEs for MSMs with weights from our calibration approach had smaller variances and MSEs than IPWEs with weights from the MLE and CBPS approaches, under correct and incorrect model specification. The flexibility and computational efficiency of our calibration approach makes it well equipped to deal with common scenarios in fitting MSMs using observational cohort data from clinical studies such as the HERS. This will hopefully promote more widespread use of MSMs for various types of treatments/exposure and outcomes in practice.

We emphasize that choosing the correct set of covariates and functionals for balancing remains important for the performance of IPWEs with the proposed approach. This is related to the challenging problem of “covariate selection” in causal inference literature (Shortreed and Ertefaie, 2017). Specifically, imposing *exact* covariate balance, as done in both the proposed approach and the CBPS, will limit the number of covariates and functionals included for balancing, which can reduce the robustness and efficiency of IPWEs if observed confounders and important predictors of the outcome are omitted (Wang and Zubizarreta, 2020). One possible solution is to allow *approximate* covariate balance such that more calibration restrictions can be included, as advocated in Wang and Zubizarreta (2020). Second, it would be useful to replace initial weights from MLE with initial weights estimated by data‐adaptive methods. This can provide some protection from severe model misspecification, and therefore reduce the possibility of large bias for IPWEs with calibrated weights. Third, as pointed out by the associate editor, it would be useful to construct double robust (DR) estimators based on the proposed calibration approach. Focusing on binary treatments, a natural way to construct a DR estimator with our CBWs is to either incorporate them into the augmented inverse probability weighted estimator, or into the targeted maximum likelihood approach as a clever covariate. However, it is not clear if such estimators will perform well even when the treatment assignment and outcome regression models are *both mildly misspecified* (Kang and Schafer, 2007). Therefore it would be desirable to extend our proposed approach by developing new DR estimators that can perform well when either, but not necessarily both, of the working models for nuisance parameters are mildly misspecified.

Similar to other CBW approaches, it warrants future research to develop sensitivity analysis strategies for the proposed approach to assess the impact of violations to the “no unmeasured confounders” assumption. Ko *et al*. (2003) implemented the sensitivity analysis approach suggested in Robins (1999a) by introducing a sensitivity parameter defined as the difference between the means of the potential outcomes given observed treatment/covariate histories. This approach is relatively straightforward for binary treatments and continuous outcomes, but less straightforward for other treatment and outcome combinations.

## Supporting information

Web appendices A–G referenced in Sections 1, 3, 4–6 and the R code for the simulation study are available with this paper at the Biometrics website on Wiley Online Library.

## Data Availability

Permission to access the HERS data can be obtained through the U.S. Centers for Disease Control and Prevention. Process to access the database and contact person can be found at https://aspe.hhs.gov/report/inventory‐federally‐sponsored‐hiv‐and‐hiv‐relevant‐databases/database‐hiv‐epidemiologic‐research‐study‐hers.
