# Supplementary material for: Joint calibrated estimation of inverse probability of treatment and censoring weights for marginal structural models
Source: Biometrics. 2020 Dec 11;78(1):115–27. doi: 10.1111/biom.13411 (PMC7612568; doi:10.1111/biom.13411)
Supplement: Supplementary file 1 — Web appendices A–G referenced in Sections 1, 3, 4–6 and the R code for the simulation study are available with this paper at the Biometrics website on Wiley Online Library. [file BIOM-78-115-s002.pdf]

Biometrics 000, 1–25 DOI: 000
000 0000

Supporting Information for "Joint Calibrated Estimation of Inverse Probability of Treatment and Censoring Weights for Marginal Structural Models" by

Sean Yiu* and Li Su**

MRC Biostatistics Unit, School of Clinical Medicine, University of Cambridge, Cambridge CB2 0SR, U.K.

*email: sean_yiu@hotmail.com

**email: li.su@mrc-bsu.cam.ac.uk

This paper has been submitted for consideration for publication in *Biometrics*

1. Web Appendix A: Related literature and our contributions

In this section, we provide a more detailed discussion of related literature and our contributions.

The literature of covariate balancing weights (CBWs) mostly focuses on binary point treatments (e.g., Graham et al., 2012; Hainmueller, 2012; Imai and Ratkovic, 2014; Zubizarreta, 2015; Chan et al., 2016; Fong et al., 2018). Imai and Ratkovic (2015) first extended CBW methods to marginal structural models (MSMs) by adapting their 'Covariate Balancing Propensity Score' (CBPS) method (Imai and Ratkovic, 2014) to time-varying binary treatments in longitudinal settings. We initially wanted to apply the CBPS method to our HERS data application. However, we soon realized that this wasn't possible because (1) CBPS can only be applied to binary treatments (whereas we have ordinal treatments in the HERS data), (2) it does not handle dependent censoring that occurred in the HERS data, and (3) it does not accommodate many covariates and visits very well. This last issue arises because, at each visit, the covariates are balanced across all possible treatment sequences at future visits, therefore the moment conditions in the CBPS increases exponentially with the number of visits. For instance, in the data example of Imai and Ratkovic (2015), 12 covariates at 5 visits require 1548 moment conditions. This makes the CBPS method computationally intensive, which hampers its use in practice. Overall, our method has the following characteristics that are distinct from the CBPS method. First, our method can be applied to time-varying treatments of arbitrary marginal distributions. In the motivating HERS data, we focus on ordinal time-varying treatments. Second, we deal with both time-varying confounding and dependent censoring that are common in longitudinal settings. Third, our method can be applied to both longitudinal outcomes over time and eventual outcomes at a study end, while the CBPS method handles eventual outcomes only. Fourth, our method can incorporate a variety of stabilized weight structures that condition on baseline covariates, while it is not

clear how to include arbitrary stabilized weight structures in the CBPS. Lastly, the number of moment conditions in our method does not have to increase exponentially with the number of follow-up visits, unlike in Imai and Ratkovic (2015). For example, they can increase linearly even if separate treatment assignment models are specified at each visit. This is the case irrespective of how we implement the weight estimation, i.e., either with Type (1) or Type (2) method. The moment conditions in our method would only increase exponentially if separate treatment assignment models are specified for *each observed treatment sequence*. In other words, our method allows for parsimony in deriving moment conditions that is necessary when there exist many covariates and visits. Along with the proposed implementation methods for calibration, this greatly facilitates the computation and practical use of our method, especially when non-parametric bootstrap is used for inference.

Recently, Yiu and Su (2018) proposed a unified framework of CBWs for general point treatments and also extended it to estimate the *short-term direct* causal effect of a time-varying treatment on a longitudinal outcome. In this paper, motivated by the challenges in the HERS data, we consider the more difficult problem of estimating the *total* causal effect of a treatment sequence, i.e., both the direct treatment effect and the indirect treatment effect through time-varying confounders, on a longitudinal outcome over time. In this case, the weight estimation procedure in Yiu and Su (2018) will not result in a consistent estimator of the total effect because it is only designed to remove confounding for estimating the direct effect of the current treatment. In contrast, our proposed method can be used to consistently estimate the total effect of a treatment sequence in MSMs of flexible forms under the same condition for the standard inverse probability weighted estimator (IPWE), i.e., the treatment assignment model is correctly specified. Our calibration approach is also computationally efficient, especially for time-varying weights in longitudinal settings, because the number of parameters to be estimated does not increase with the number of observations unlike

in Yiu and Su (2018). Furthermore, we introduce new covariate balancing moment conditions for dependent censoring. Unlike inverse probability of treatment weighting (IPTW), which balances covariates across treatment groups, inverse probability of censoring weighting (IPCW) intends to create a representative sample of the target population (in the absence of censoring) in terms of observed covariates that predict the censoring process. Therefore, the distinction between objectives of IPTW and IPCW requires different covariate balancing conditions for handling time-varying confounding and dependent censoring respectively.

Kallus and Santacatterina (2019) also proposed a CBW approach for MSMs with binary treatments in the presence of time-varying confounding and dependent censoring. Specifically, they use kernel smoothing to flexibly model expectations of a potential outcome at a study end, conditional on treatment and covariate histories up to each visit in the follow-up. Then weights are estimated by minimizing an upper bound for imbalances of time-varying variables (as characterized by conditional expectations of the potential outcome with kernels) over time plus some penalty for the variability of the weights. Because this approach uses information from the observed outcome when modeling conditional expectations of the potential outcome, it is distinct from the standard IPTW approach and our calibrated approach for CBW in MSMs, where only information for the treatment process is used. In addition, the approach in Kallus and Santacatterina (2019) involves tuning hyperparameters of the kernels and the penalization parameter for weight estimation. It is also not clear how to generalize their method to accommodate continuous and other non-binary treatments over time, which is one of the main motivations for developing our method.

Zhou and Wodtke (2020) took a different approach from the aforementioned methods. Instead of modeling the treatment assignments and/or potential outcomes over time, they proposed to model the conditional expectations of the time-varying confounders (including the functional form and interactions of the distinct confounders) that are affected by

previous treatments, given the treatment and covariate histories. In particular, they used these models to compute the residuals for the time-varying confounders, and then they estimated covariate balancing weights by setting the weighted correlation between these residuals and future treatments, as well as the variables in the conditional expectation models for time-varying confounders, to zero. Because this 'residual balancing' approach of Zhou and Wodtke (2020) does not require modeling the treatment assignments, it is applicable to non-binary treatments. However, the requirement of correctly modeling conditional expectations of the (possibly high-dimensional) time-varying confounders can be extremely burdensome in practice, compared with the approaches focusing on the treatment model, including ours. In addition, dependent censoring and longitudinal outcomes are not accommodated in Zhou and Wodtke (2020), either.

In the context of handling dependent censoring only, Han (2016) proposed a calibrated estimation approach for weights in IPCW. Specifically, Han (2016) proposed to calibrate inverse probability of censoring weights by imposing similar restrictions to (13) in the main text. However, the focus of Han (2016) was on an eventual outcome at the end of study, Y_{iT}. For comparison, we derive restrictions for his target of inference $\mathrm{E}(Y_{iT})$ as

$$\sum_{j=1}^{T}\sum_{i=1}^{n}[R_{ij}W_{ij}^{C\star}(\boldsymbol{\lambda}) - R_{i,j-1}W_{i,j-1}^{C\star}(\boldsymbol{\lambda})]\widetilde{\boldsymbol{H}}_{i,j-1} = \mathbf{0}, \tag{1}$$

where $W_{ij}^{C\star}(\boldsymbol{\lambda})$ is the calibrated weight for IPCW and $\boldsymbol{\lambda}$ is the parameter vector to be estimated for the calibration. The restrictions in (1) are based on (8) of the main text but with T as the upper limit of the product in k. If $\widetilde{\boldsymbol{H}}_{i,j-1}$ includes baseline covariates $\boldsymbol{V}_i$, these restrictions impose

$$\sum_{i=1}^{n} R_{iT}W_{iT}^{C\star}(\boldsymbol{\lambda}) = n \quad \text{and} \quad \sum_{i=1}^{n} R_{iT}W_{iT}^{C\star}(\boldsymbol{\lambda})\boldsymbol{V}_i = \sum_{i=1}^{n}\boldsymbol{V}_i,$$

i.e., at visit T the sample size after weighting is n and the weighted average of $\boldsymbol{V}_i$ is equal to the sample average of $\boldsymbol{V}_i$.

The restrictions in (1) and those in Han (2016) differ in the way they achieve parsimony,

which helps prevent unstable weights. In particular, Han (2016) imposes separate restrictions at each visit, by multiplying the summands in (1) by the visit indicator $I(j = k)$ $(k = 1, \ldots, T)$. This is feasible with few follow-up visits, e.g., the simulation study in Han (2016) has three visits. Our approach leaves the degree of smoothing over time at the discretion of the researcher. For example, natural cubic splines can be incorporated to reflect non-linear time trend in the censoring process, similarly to the approach in Hernán et al. (2001) for approximating the time-varying baseline treatment assignment distribution. Han (2016) also uses multiple estimates of $\mathrm{E}(Y_{iT}|\overline{H}_{i,j-1})$ based on different working models in place of $\widetilde{\boldsymbol{H}}_{i,j-1}$ in (1). Including more estimates of $\mathrm{E}(Y_{iT}|\overline{H}_{i,j-1})$ in the restrictions generally lead to an increase in efficiency when all working models are misspecified (Han, 2016). In this regard, Han's approach is more parsimonious than ours because his restrictions are contained in (1) if his working models for $\mathrm{E}(Y_{iT}|\overline{H}_{i,j-1})$ are linear in $\widetilde{\boldsymbol{H}}_{i,j-1}$.

2. Web Appendix B: Deriving calibration restrictions for treatment assignment in the eventual outcome setting

For the setting with an eventual outcome at visit T (e.g., the CD4 count at the study end of the HERS), restrictions for the stabilized inverse probability of treatment weights can be derived by using the same procedure as in Section 4.1 of the main text. However, since in this case we would only need to obtain calibrated weights at visit T, $W_{iT}^{AC\star}(\boldsymbol{\lambda})$, (4) of the main text will only contain the terms weighted by $W_{iT}^{AC\star}(\boldsymbol{\lambda})$, i.e., the terms where $j = T$ in (4) of the main text. This results in the restriction

$$\sum_{i=1}^{n} R_{iT} W_{iT}^{AC\star}(\boldsymbol{\lambda}) \sum_{k=1}^{T} \frac{\partial}{\partial \boldsymbol{\beta}_{\mathrm{w}}} \log\{\mathrm{pr}(A_{ik} \mid \overline{X}_{i,k-1}; \boldsymbol{\beta}_{\mathrm{w}})\}\Big|_{\{\boldsymbol{\beta}_{\mathrm{wb}}=\hat{\boldsymbol{\alpha}}, \boldsymbol{\beta}_{\mathrm{wd}}=\mathbf{0}\}} = \mathbf{0}.$$

For implementation by the Type (1) method, a normalizing restriction would again need to be imposed if only IPTW is to be applied. For example,

$$\sum_{i=1}^{n} R_{iT} W_{iT}^{A\star}(\boldsymbol{\lambda}) = \sum_{i=1}^{n} R_{iT}.$$

3. Web Appendix C: Calibration restrictions for censoring

3.1 *Inductive proof of the validity of IPCW*

In this section, we prove the proposition that at visit j ($j = 1, \ldots, T$), IPCW creates a representative sample of the target population (in the absence of censoring) at visit j after weighting the uncensored observations by W_{ij}^C.

Proof. Our proof follows by induction. Let $W_{ij}^C = \prod_{k=1}^{j} 1/\mathrm{pr}(R_{ik} = 1 \mid \overline{H}_{i,k-1}, R_{i,k-1} = 1)$. First, we prove the base case (i.e., $j = 1$). This will require showing that $\mathrm{pr}^*(R_{i1} \mid \overline{H}_{i0}) = 1/2$, where $*$ denotes the pseudo-population consisting of the $W_{i1}^C - 1$ copies of the uncensored observations (i.e., those with $R_{i1} = 1$) and the censored observations (i.e., those with $R_{i1} = 0$).

$$\begin{aligned}
\mathrm{pr}^*(R_{i1} \mid \overline{H}_{i0}) &= \frac{R_{i1}\{1/\mathrm{pr}(R_{i1} = 1 \mid \overline{H}_{i0}) - 1\}\mathrm{pr}(R_{i1} = 1 \mid \overline{H}_{i0}) + (1 - R_{i1})\mathrm{pr}(R_{i1} = 0 \mid \overline{H}_{i0})}{\{1/\mathrm{pr}(R_{i1} = 1 \mid \overline{H}_{i0}) - 1\}\mathrm{pr}(R_{i1} = 1 \mid \overline{H}_{i0}) + \mathrm{pr}(R_{i1} = 0 \mid \overline{H}_{i0})} \\
&= \frac{R_{i1}\mathrm{pr}(R_{i1} = 0 \mid \overline{H}_{i0}) + (1 - R_{i1})\mathrm{pr}(R_{i1} = 0 \mid \overline{H}_{i0})}{2\mathrm{pr}(R_{i1} = 0 \mid \overline{H}_{i0})} \\
&= 1/2.
\end{aligned}$$

The base case now follows because we have shown that the pseudo-population formed by $W_{i1}^C - 1$ copies of the uncensored observations is a representative sample of the censored observations. Thus including an additional copy of the uncensored observations in the pseudo-population (i.e., the total $\sum_{i=1}^{n} W_{i1}^C$ copies of the uncensored observations) will create a representative sample of the target population at visit 1.

Assuming the proposition at visit $j-1$ holds, i.e., the pseudo-population formed by weighting the uncensored observations at visit $j-1$ by $W_{i,j-1}^C$ is a representative sample of the target population at visit $j-1$. Denote this pseudo-population at visit $j-1$ by the new population. The inductive step requires showing that weighting the uncensored observations of this new population by $1/\mathrm{pr}(R_{ij} = 1 \mid \overline{H}_{i,j-1}, R_{i,j-1} = 1)$ creates a representative sample of the target population at visit j. This would then imply the proposition holds at visit j because weighting

the uncensored observations at visit j by $W^C_{i,j-1} \times 1/\mathrm{pr}(R_{ij} = 1 \mid \overline{H}_{i,j-1}, R_{i,j-1} = 1) = W^C_{ij}$ creates a representative sample of the target population at visit j.

The proof is completed by recognizing that the inductive steps can be proved in the same way as the base case but where the new population is now taken to be the baseline population (i.e., at visit 0).

3.2 *Equivalence of censoring restrictions in (12) and (13) of the main text*

By writing out the terms in the summation in j, equation (12) of the main text is equal to

$$\sum_{i=1}^{n} \Big[T\{R_{i1}W^{AC\star}_{i1}(\boldsymbol{\lambda}) - 1\}\widetilde{\boldsymbol{H}}_{i0} + (T-1)\{R_{i2}W^{AC\star}_{i2}(\boldsymbol{\lambda}) - R_{i1}W^{AC\star}_{i1}(\boldsymbol{\lambda})\}\widetilde{\boldsymbol{H}}_{i1} + \ldots + \{R_{iT}W^{AC\star}_{iT}(\boldsymbol{\lambda}) - R_{i,T-1}W^{AC\star}_{i,T-1}(\boldsymbol{\lambda})\}\widetilde{\boldsymbol{H}}_{i,T-1}\Big] = \mathbf{0},$$

which follows from the fact that $R_{i0} = 1$ for all i and by convention $W^{AC\star}_{i0}(\boldsymbol{\lambda}) = 1$ for all i. Next, we bring $T\sum_{i=1}^{n} \widetilde{\boldsymbol{H}}_{i0}$ to the right-hand side,

$$\sum_{i=1}^{n} \Big[TR_{i1}W^{AC\star}_{i1}(\boldsymbol{\lambda})\widetilde{\boldsymbol{H}}_{i0} + (T-1)\{R_{i2}W^{AC\star}_{i2}(\boldsymbol{\lambda}) - R_{i1}W^{AC\star}_{i1}(\boldsymbol{\lambda})\}\widetilde{\boldsymbol{H}}_{i1} + \ldots + \{R_{iT}W^{AC\star}_{iT}(\boldsymbol{\lambda}) - R_{i,T-1}W^{AC\star}_{i,T-1}(\boldsymbol{\lambda})\}\widetilde{\boldsymbol{H}}_{i,T-1}\Big] = T\sum_{i=1}^{n}\widetilde{\boldsymbol{H}}_{i0}.$$

Now collect the coefficients for the weights

$$\sum_{i=1}^{n} \Big[R_{i1}W^{AC\star}_{i1}(\boldsymbol{\lambda})\{T\widetilde{\boldsymbol{H}}_{i0} - (T-1)\widetilde{\boldsymbol{H}}_{i1}\} + R_{i2}W^{AC\star}_{i2}(\boldsymbol{\lambda})\{(T-1)\widetilde{\boldsymbol{H}}_{i1} - (T-2)\widetilde{\boldsymbol{H}}_{i2}\} + \ldots + R_{iT}W^{AC\star}_{iT}(\boldsymbol{\lambda})\widetilde{\boldsymbol{H}}_{i,T-1}\Big] = T\sum_{i=1}^{n}\widetilde{\boldsymbol{H}}_{i0}.$$

When written in summation form, the above equation is equivalent to (13) in the main text.

3.3 *Calibration restrictions for stabilized inverse probability of censoring weights*

Similarly to inverse probability of treatment weighting, stabilized weights for censoring, $SW^C_{ij}(\hat{\boldsymbol{\eta}}, \hat{\boldsymbol{\theta}}) = W^C_{ij}(\hat{\boldsymbol{\theta}}) \prod_{k=1}^{j} \pi^{\mathrm{s}}_{ik}(\hat{\boldsymbol{\eta}})$, can be used, where $\pi^{\mathrm{s}}_{ik}(\boldsymbol{\eta}) = \mathrm{pr}(R_{ik} = 1 \mid \overline{A}_{i,k-1}, R_{i,k-1} = 1; \boldsymbol{\eta})$ is a parametric model for the censoring process given the treatment history only.

For completeness, we describe our proposed method for calibrating stabilized weights for censoring in this section.

The idea of stabilized weights for visit j is to create a pseudo-population that is representative of the population that would have been observed had nobody been censored up to and including visit j, which we refer to as the complete population, and where each patient in the complete population has been weighted by $\prod_{k=1}^{j} \pi_{ik}^{\mathrm{s}}(\hat{\boldsymbol{\eta}})$. In other words, we down-weight patients who are unlikely to be observed at visit j based solely on treatment history. Therefore the task is less ambitious than what unstabilized weights are trying to achieve. Restrictions for the stabilized weights can be derived, e.g., by multiplying the summands indexed by j in equation (12) of the main text by the stabilizing factors $\prod_{k=1}^{j} \pi_{ik}^{\mathrm{s}}(\hat{\boldsymbol{\eta}})$,

$$\sum_{j=1}^{T}\sum_{i=1}^{n}(T-j+1)\left[R_{ij}SW_{ij}^{AC\star}(\boldsymbol{\lambda}) - R_{i,j-1}SW_{i,j-1}^{AC\star}(\boldsymbol{\lambda})\pi_{ij}^{\mathrm{s}}(\hat{\boldsymbol{\eta}})\right]\widetilde{\boldsymbol{H}}_{i,j-1} = \mathbf{0},$$

where $SW_{ij}^{AC\star}(\boldsymbol{\lambda})$ are new the calibrated weights containing the stabilizing factors $\prod_{k=1}^{j} \pi_{ik}^{\mathrm{s}}(\hat{\boldsymbol{\eta}})$. Specifically, for the Type (1) method, $SW_{ij}^{AC\star}(\boldsymbol{\lambda}) = SW_{ij}^{A}(\hat{\boldsymbol{\alpha}}, \hat{\boldsymbol{\beta}})SW_{ij}^{C}(\hat{\boldsymbol{\eta}}, \hat{\boldsymbol{\theta}})c(\overline{X}_{ij}, \overline{H}_{i,j-1}, \boldsymbol{\lambda})$, and for implementation by the Type (2) method, $SW_{ij}^{AC\star}(\boldsymbol{\lambda}) = SW_{ij}^{A}(\hat{\boldsymbol{\alpha}}, \boldsymbol{\beta})SW_{ij}^{C}(\hat{\boldsymbol{\eta}}, \boldsymbol{\theta})$, where $\boldsymbol{\lambda} = \{\boldsymbol{\beta}, \boldsymbol{\theta}\}$ are calibration parameters to be estimated. Note that these restrictions, after scaling by n, are satisfied asymptotically ($n \to \infty$) if the treatment and censoring process models are correctly specified.

4. Web Appendix D: Conditions for models for deriving calibration restrictions to produce consistent estimators of MSM parameters

The standard MLE approach to weight estimation will result in consistent IPWEs of MSM parameters if the treatment assignment model $\mathrm{pr}(A_{ij} \mid \overline{X}_{i,j-1}; \boldsymbol{\beta})$ and model for the censoring process $\mathrm{pr}(R_{ij} = 1 \mid \overline{H}_{i,j-1}, R_{i,j-1} = 1; \boldsymbol{\theta})$ are correctly specified at each visit. This remains true with our joint calibration approach if, in addition, the models $\mathrm{pr}(A_{ij} \mid \overline{X}_{i,j-1}; \boldsymbol{\beta}_{\mathrm{w}})$ and $\mathrm{pr}(R_{ij} = 1 \mid \overline{H}_{i,j-1}, R_{i,j-1} = 1; \boldsymbol{\theta}_{\mathrm{w}})$, which are used to derive restrictions (4) and (11) of

the main text, are parameterized such that $\text{pr}(A_{ij} \mid \overline{X}_{i,j-1}; \boldsymbol{\beta}_{\text{wb}} = \hat{\boldsymbol{\alpha}}, \boldsymbol{\beta}_{\text{wd}} = \mathbf{0}) = \text{pr}(A_{ij} \mid \overline{A}_{i,j-1}; \hat{\boldsymbol{\alpha}})$ (here $\text{pr}(A_{ij} \mid \overline{A}_{i,j-1}; \hat{\boldsymbol{\alpha}})$ are the terms in the numerator of stabilized treatment weights by MLE) and $\text{pr}(R_{ij} = 1 \mid \overline{H}_{i,j-1}, R_{i,j-1} = 1; \boldsymbol{\theta}_{\text{w}} = \mathbf{0}) = 1/2$, respectively. Therefore, we recommend choosing treatment process models for deriving restrictions in which the model for the numerator of the initial stabilized weights is nested, while logistic models with the restrictions in (12) of the main text suffice for the censoring process.

In the next section, we give a heuristic argument for why our joint calibration approach will result in consistent IPWEs for MSM parameters under the aforementioned conditions. In particular, we show that the true inverse probability of treatment weights satisfy the restrictions (4) of the main text asymptotically, so the calibrated weights will converge to the true weights under the aforementioned conditions. A similar argument can be provided for inverse probability of censoring weights and restrictions (11) of the main text.

4.1 *Proof that the true inverse probability of treatment weights satisfy the proposed moment conditions asymptotically*

Without loss of generality, suppose that $\overline{A}_{ij}$ and $\overline{X}_{i,j-1}$ $(j = 1, \ldots, T)$ are continuous. Let $SW_{ij}^{A}(\boldsymbol{\alpha}) = \prod_{k=1}^{j} \text{pr}(A_{ik} \mid \overline{A}_{i,k-1}; \boldsymbol{\alpha}) / \prod_{k=1}^{j} \text{pr}(A_{ik} \mid \overline{X}_{i,k-1})$. That is, the numerator in $SW_{ij}^{A}(\boldsymbol{\alpha})$ is arbitrary and only depends on treatment history, and the denominator in $SW_{ij}^{A}(\boldsymbol{\alpha})$ contains the true treatment assignment probabilities. We show that

$$\text{E}\left[SW_{ij}^{A}(\boldsymbol{\alpha}) \sum_{k=1}^{j} \frac{\partial}{\partial \boldsymbol{\beta}_{\text{w}}} \log\{\text{pr}(A_{ik} \mid \overline{X}_{i,k-1}; \boldsymbol{\beta}_{\text{w}})\} \Big|_{\{\boldsymbol{\beta}_{\text{wb}} = \boldsymbol{\alpha}, \boldsymbol{\beta}_{\text{wd}} = \mathbf{0}\}} \right] = \mathbf{0}$$

for any model $\text{pr}(A_{ik} \mid \overline{X}_{i,k-1}; \boldsymbol{\beta}_{\text{w}})$ that satisfies $\text{pr}(A_{ik} \mid \overline{X}_{i,k-1}; \boldsymbol{\beta}_{\text{wb}} = \boldsymbol{\alpha}, \boldsymbol{\beta}_{\text{wd}} = \mathbf{0}) = \text{pr}(A_{ik} \mid \overline{A}_{i,k-1}; \boldsymbol{\alpha})$.

Proof.

$$
\begin{aligned}
&\mathrm{E}\left[SW_{ij}^{A}(\boldsymbol{\alpha})\sum_{k=1}^{j}\frac{\partial}{\partial\boldsymbol{\beta}_{\mathrm{w}}}\log\{\mathrm{pr}(A_{ik}\mid \overline{X}_{i,k-1};\boldsymbol{\beta}_{\mathrm{w}})\}\Big|_{\{\boldsymbol{\beta}_{\mathrm{wb}}=\boldsymbol{\alpha},\boldsymbol{\beta}_{\mathrm{wd}}=\mathbf{0}\}}\right]\\
=&\mathrm{E}\left[\prod_{k=1}^{j}\frac{\mathrm{pr}(A_{ik}\mid \overline{A}_{i,k-1};\boldsymbol{\alpha})}{\mathrm{pr}(A_{ik}\mid \overline{X}_{i,k-1})}\left\{\frac{\partial}{\partial\boldsymbol{\beta}_{\mathrm{w}}}\log\left\{\prod_{k=1}^{j}\mathrm{pr}(A_{ik}\mid \overline{X}_{i,k-1};\boldsymbol{\beta}_{\mathrm{w}})\right\}\Big|_{\{\boldsymbol{\beta}_{\mathrm{wb}}=\boldsymbol{\alpha},\boldsymbol{\beta}_{\mathrm{wd}}=\mathbf{0}\}}\right\}\right]\\
=&\mathrm{E}\left[\prod_{k=1}^{j}\frac{1}{\mathrm{pr}(A_{ik}\mid \overline{X}_{i,k-1})}\left\{\frac{\partial}{\partial\boldsymbol{\beta}_{\mathrm{w}}}\prod_{k=1}^{j}\mathrm{pr}(A_{ik}\mid \overline{X}_{i,k-1};\boldsymbol{\beta}_{\mathrm{w}})\Big|_{\{\boldsymbol{\beta}_{\mathrm{wb}}=\boldsymbol{\alpha},\boldsymbol{\beta}_{\mathrm{wd}}=\mathbf{0}\}}\right\}\right]\\
=&\int_{\overline{A}_{ij}}\int_{\overline{X}_{i,j-1}}\prod_{k=1}^{j}\frac{1}{\mathrm{pr}(A_{ik}\mid \overline{X}_{i,k-1})}\left\{\frac{\partial}{\partial\boldsymbol{\beta}_{\mathrm{w}}}\prod_{k=1}^{j}\mathrm{pr}(A_{ik}\mid \overline{X}_{i,k-1};\boldsymbol{\beta}_{\mathrm{w}})\Big|_{\{\boldsymbol{\beta}_{\mathrm{wb}}=\boldsymbol{\alpha},\boldsymbol{\beta}_{\mathrm{wd}}=\mathbf{0}\}}\right\}\\
&\times\prod_{k=1}^{j}\left\{\mathrm{pr}(A_{ik}\mid \overline{X}_{i,k-1})\mathrm{pr}(X_{i,k-1}\mid \overline{A}_{i,k-1},\overline{X}_{i,k-2})\right\}\,d\overline{A}_{ij}\,d\overline{X}_{i,j-1}\\
=&\int_{\overline{A}_{ij}}\int_{\overline{X}_{i,j-1}}\left\{\frac{\partial}{\partial\boldsymbol{\beta}_{\mathrm{w}}}\prod_{k=1}^{j}\mathrm{pr}(A_{ik}\mid \overline{X}_{i,k-1};\boldsymbol{\beta}_{\mathrm{w}})\Big|_{\{\boldsymbol{\beta}_{\mathrm{wb}}=\boldsymbol{\alpha},\boldsymbol{\beta}_{\mathrm{wd}}=\mathbf{0}\}}\right\}\\
&\times\prod_{k=1}^{j}\mathrm{pr}(X_{i,k-1}\mid \overline{A}_{i,k-1},\overline{X}_{i,k-2})\,d\overline{A}_{ij}\,d\overline{X}_{i,j-1}.
\end{aligned}
$$

Note that the aforementioned condition on the treatment process models for deriving restrictions $\mathrm{pr}(A_{ij}\mid \overline{X}_{i,j-1};\boldsymbol{\beta}_{\mathrm{wb}}=\boldsymbol{\alpha},\boldsymbol{\beta}_{\mathrm{wd}}=\mathbf{0})=\mathrm{pr}(A_{ij}\mid \overline{A}_{i,j-1};\boldsymbol{\alpha})$ was used in the third line. Next, assuming that we can interchange differentiation and integration, the above expression is equal to

$$
\begin{aligned}
&\frac{\partial}{\partial\boldsymbol{\beta}_{\mathrm{w}}}\left\{\int_{\overline{A}_{ij}}\int_{\overline{X}_{i,j-1}}\prod_{k=1}^{j}\mathrm{pr}(A_{ik}\mid \overline{X}_{i,k-1};\boldsymbol{\beta}_{\mathrm{w}})\mathrm{pr}(X_{i,k-1}\mid \overline{A}_{i,k-1},\overline{X}_{i,k-2})\,d\overline{A}_{ij}\,d\overline{X}_{i,j-1}\right\}\Big|_{\{\boldsymbol{\beta}_{\mathrm{wb}}=\boldsymbol{\alpha},\boldsymbol{\beta}_{\mathrm{wd}}=\mathbf{0}\}}\\
=&\frac{\partial}{\partial\boldsymbol{\beta}_{\mathrm{w}}}1\Big|_{\{\boldsymbol{\beta}_{\mathrm{wb}}=\boldsymbol{\alpha},\boldsymbol{\beta}_{\mathrm{wd}}=\mathbf{0}\}}=\mathbf{0}.
\end{aligned}
$$

5. Web Appendix E: Simulation

We conduct simulation studies to assess the finite sample performance of the IPWE for MSMs based on our calibration approach, in comparison with the MLE and CBPS approaches. The R code for the simulation studies is also available as separate files in the Supporting Information.

5.1 *Design*

The design of the first simulation study is motivated by the HERS data, where the time-varying treatment is an ordinal variable. Because the CBPS approach can only handle binary time-varying treatments with a small number of visits in the absence of censoring, we are only able to compare the performance of our calibration approach with the MLE approach in the first simulation study. To include the CBPS approach for comparison, we also design the second simulation study with binary treatments at a few follow-up visits.

5.1.1 *The first simulation study.* The data generating mechanism for a patient in the first simulation study is summarized in Table 1, and Figure 1 provides a pictorial description. We omit the subscript i for patients for clearer presentation.

[Table 1 about here.]

[Figure 1 about here.]

In this set-up there are four time-varying confounders $\{X_{j-1,1}, X_{j-1,2}, X_{j-1,3}, X_{j-1,4}\}$ ($j = 1, \ldots, 10$), which affect the treatment assignment $\{A_j^0, A_j^1\}$ and the mean of the outcome $\mathrm{E}(Y_j)$ at visit j. In addition, the variances of $\{X_{j-1,1}, X_{j-1,2}\}$ and the means of $\{X_{j-1,3}, X_{j-1,4}\}$ are affected by treatment history $\overline{A}_{j-1}$. Time-varying confounding arises from $\{X_{j-1,3}, X_{j-1,4}\}$ because conditioning on them via regression adjustment blocks the effect of previous treatments (i.e., $\sum_{t=0}^{j-1}(A_t^0 + A_t^1)$) through themselves.

For the censoring process, we specify two scenarios. In Scenario 1, no censoring occurs as $\mathrm{pr}(R_j \mid R_{j-1} = 1) = 1$. In Scenario 2, covariate-dependent censoring occurs and selection bias is induced because $\mathrm{E}(Y_j)$ depends on $X_{j-1,l}$ and $\mathrm{E}(X_{j-1,l} \mid \overline{A}_{j-1}, R_j = 1) \neq \mathrm{E}(X_{j-1,l}|\overline{A}_{j-1})$ ($l = 1, \ldots, 4$).

We assume a MSM $\mathrm{E}(Y_j^{\overline{a}_j}) = \gamma_0 + \gamma_1 \sum_{t=0}^{j}(a_t^0 - a_t^1) + \gamma_2 \sum_{t=0}^{j} a_t^1$, where a_t^0 and a_t^1 are

potential values of treatment indicators A_t^0 and A_t^1. The true treatment effects can be derived by noting that $\mathrm{E}(Y_j|\overline{A}_j) = 200 + 10\sum_{t=0}^{j}(A_t^0 + A_t^1)$, and thus $\gamma_1 = 10$ and $\gamma_2 = 20$.

Data from each patient are generated independently. We simulate 2500 data sets with 500, 1000 and 2500 patients and 10 scheduled follow-up visits after baseline. For weight estimation, we assume the logistic models for the treatment indicators described in Section 4.1.1 of the main text and the logistic model for the censoring process described in Section 4.2 of the main text. In Scenario 1, we include an intercept and the main effects of $\{A_{j-1}^0, A_{j-1}^1\}$ in the logistic models for the numerator of the stabilized treatment weights at visit j. For the denominator, we additionally include the main effects of $\{X_{j-1,1}, X_{j-1,2}, X_{j-1,3}, X_{j-1,4}\}$ to ensure that the treatment assignment models are correctly specified. In Scenario 2, we use the same treatment assignment models as Scenario 1, and include visit-specific indicators and the main effects of $\{A_{j-1}^0, A_{j-1}^1, X_{j-1,1}, X_{j-1,2}, X_{j-1,3}, X_{j-1,4}\}$ in the censoring model at visit j. The visit-specific intercepts were included to impose analogous restrictions to (7) of the main text in Scenario 2. To consider a functional form misspecification, we use a set of transformed covariates $\{X_{j-1,1}^t, X_{j-1,2}^t, X_{j-1,3}^t, X_{j-1,4}^t\}$ of the form $X_{j-1,1}^t = (X_{j-1,1})^3/9$, $X_{j-1,2}^t = X_{j-1,1}X_{j-1,2}$, $X_{j-1,3}^t = \log(|X_{j-1,3}|)+4$ and $X_{j-1,4}^t = 1/\{1+\exp(X_{j-1,4})\}$ in place of the correct covariates $\{X_{j-1,1}, X_{j-1,2}, X_{j-1,3}, X_{j-1,4}\}$ for deriving restrictions for calibration and for estimating the initial weights (Type (1) method only).

We apply both the MLE approach and the proposed calibration approach (implemented by both Type (1) and (2) methods) to estimate the weights for IPTW and IPTCW. The weights by MLE are scaled to sum to the number of observations that would have been obtained if no censoring occurs, similarly to Cao et al. (2009). This scaling step will help to bound extreme weights from MLE. For implementation by the Type (1) method, we use the weights estimated by MLE as the initial weights. We then apply IPTW for Scenario 1 and

IPTCW for Scenario 2 with the estimated weights and use the estimating equations in (1) of the main text to estimate γ_1 and γ_2.

5.1.2 *The second simulation study.* The data generating mechanism of the second simulation study, summarized in Table 2, is very similar to that of the first simulation study except that binary treatments at five follow-up visits are included and no censoring occurs.

[Table 2 about here.]

We assume a MSM $\mathrm{E}(Y_j^{\overline{a}_j}) = \gamma_0 + \gamma_1 \sum_{t=0}^{j} a_t$, where a_t is the potential value of a binary treatment indicator A_t. The true treatment effects can be derived by noting that $\mathrm{E}(Y_j|\overline{A}_j) = 200 + 10\sum_{t=0}^{j} A_t$, and thus $\gamma_1 = 10$.

Data from each patient are generated independently. We simulate 2500 data sets with 500, 1000 and 2500 patients and 5 scheduled follow-up visits after baseline. For weight estimation, we assume a logistic model for the binary treatment indicator (similar to those in Section 4.1.1 of the main text) and the logistic model for the censoring process described in Section 4.2 of the main text. We include an intercept and the main effect of A_{j-1} in the logistic model for the numerator of the stabilized treatment weights at visit j. For the denominator, we additionally include the main effects of $\{X_{j-1,1}, X_{j-1,2}, X_{j-1,3}, X_{j-1,4}\}$ to ensure that the treatment assignment models are correctly specified. To consider a functional form misspecification, we use a set of transformed covariates $\{X^t_{j-1,1}, X^t_{j-1,2}, X^t_{j-1,3}, X^t_{j-1,4}\}$ of the form $X^t_{j-1,1} = (X_{j-1,1})^3/9$, $X^t_{j-1,2} = X_{j-1,1}X_{j-1,2}$, $X^t_{j-1,3} = \log(|X_{j-1,3}|) + 4$ and $X^t_{j-1,4} = 1/\{1+\exp(X_{j-1,4})\}$ in place of the correct covariates $\{X_{j-1,1}, X_{j-1,2}, X_{j-1,3}, X_{j-1,4}\}$ for the CBPS approach, for deriving restrictions for calibration and for estimating the initial weights (Type (1) method only).

We apply the MLE approach, the proposed calibration approach (implemented by both Type (1) and (2) methods) and the CBPS approach by Imai and Ratkovic (2015) to estimate the weights for IPTW. The weights by MLE are scaled to sum to the total number of

observations. For implementation by the Type (1) method, we use the weights estimated by MLE as the initial weights. We use the R package CBPS to implement the CBPS approach. Because CBPS can only deal with eventual outcomes, we have to estimate the weights at each of the 5 visits separately for the longitudinal outcomes in the simulated datasets. The two-step estimator with the low-rank approximation of the variance described in Imai and Ratkovic (2014) are used to speed up the computation of CBPS. We then apply IPTW with the estimated weights and use the estimating equations in (1) of the main text to estimate γ_1.

5.2 *Results*

5.2.1 *Comparison of the MLE and calibration approaches in the first simulation study.* Table 3 summarizes the results of the first simulation study. When the models for the treatment and censoring processes are correctly specified, i.e., when the correct covariates are used, it is not surprising that the biases from IPWEs with weights from MLE and with calibrated weights are negligible and the standard deviations decrease as sample size increases. However, the IPWEs with calibrated weights have smaller standard deviations and mean squared errors than the IPWEs with weights from MLE.

[Table 3 about here.]

In contrast, when the models for the treatment and censoring processes are misspecified, i.e., when the transformed covariates are used, the IPWEs with weights from MLE and with calibrated weights both have non-negligible biases that do not decrease with increasing sample size, although for most of the scenarios, the IPWEs with calibrated weights have slightly smaller biases. However, the IPWEs with calibrated weights are more efficient with much smaller standard deviations. As a result, the IPWEs with calibrated weights have much smaller mean squared errors than the IPWEs with weights from MLE.

A more alarming feature of the IPWEs with weights from MLE is that the large standard

deviations and mean squared errors even increase with sample size. Under model misspecification, this occurs because a few sets of estimated weights from maximum likelihood exacerbate the extremeness of the tails of the sampling distribution of the parameter estimators as the sample size increases. Robins et al. (2007, pp. 553–4) give more details of this phenomenon. In contrast, the IPWEs with calibrated weights do not exhibit this undesirable property and show more robustness to the functional form misspecification in the set-up of the first simulation study.

Overall, these simulation results show that the proposed calibration approach can improve the efficiency and stability of the IPWEs for MSMs.

5.2.2 *Comparison of the two implementation methods for calibration in the first simulation study.* Regarding the two methods for implementing the calibration approach, it turns out that the implementation with the Type (2) method is only feasible for Scenario 1 with no censoring. In Scenario 1, the results are very similar for both Type (1) and Type (2) methods when the treatment assignment model is correctly specified. Under model misspecification with large sample sizes (n=1000, 2500), the Type (2) method appears to perform better than the Type (1) method in terms of standard deviation and mean squared error. This is likely due to the Type (1) method inheriting the poor performance of the initial weights. However, in Scenario 2 with additional dependent censoring, it was not possible to apply the Type (2) method to the majority of simulated datasets due to non-convergence. This is possibly due to that many more calibration restrictions are specified in Scenario 2 than in Scenario 1 to accommodate dependent censoring.

5.2.3 *The second simulation study.* In the second simulation study, there are considerable differences in terms of computing time between the MLE, calibration and CBPS approaches. On a Linux machine with 3.40GHz CPU and 16GB memory, it takes 0.08, 0.15, 0.25 and 202.41 seconds to estimate weights for one simulated dataset with $n = 1000$ under correct

and incorrect model misspecification, using the MLE approach, the calibration approach with Type (1) method, the calibration approach with Type (2) method and the CBPS approach, respectively. In other words, the computing time for the CBPS approach implemented by the `CBPS` package is 800 or more times than those for the calibration and MLE approaches. Specifically, the CBPS approach is considerably slower when estimating weights for the 5th follow-up visit than for the earlier visits because the number of moment conditions exponentially increases with the number of visits. In addition, as the weights at each visit are estimated separately, this also makes the computing time accumulate for the CBPS approach. As a result, we parallel the simulations using the `parallel` package in `R` on high performance clusters.

Table 4 summarizes the results of the second simulation study. When the treatment model is correctly specified, i.e., when the correct covariates are used, the biases of the IPWEs with weights from the CBPS approach are the largest, while the biases of IPWEs with weights from MLE and with calibrated weights are negligible. The CBPS approach also results in IPWEs with the largest standard deviations among all the approaches. This is likely due to that the weights from the CBPS approach have to be estimated separately at each follow-up visit, which increases the variability of the corresponding IPWEs. As a result, the IPWEs based on the CBPS approach also have the largest median absolute errors and mean squared errors.

[Table 4 about here.]

When the treatment model is misspecified, i.e., when the transformed covariates are used, the IPWEs based on all approaches have non-negligible biases. The IPWEs based on the MLE and CBPS approaches have slightly smaller biases than their counterparts from the calibration approach. However, standard deviations of the IPWEs based on the MLE and CBPS approaches are much larger than those from the calibration approach. This is especially

prominent for the MLE approach, which has been demonstrated previously in the first simulation study. As a result, the median absolute errors and mean squared errors of the IPWEs from the MLE and CBPS approaches are larger than those from the calibration approach. Note that, unlike the MLE approach, standard deviations and mean squared errors of the IPWEs based on the CBPS approach do decrease as the sample size increases. It is often conjectured that the poor performance of the MLE approach and, to a lesser extent, the CBPS approach is primarily driven by a few extreme simulations. Nevertheless, our simulation results w.r.t. the median absolute error, which is is more robust to extreme simulations than the mean squared error, also suggest that even after throwing away the worst half of the simulation results, the proposed calibration approach still outperforms the MLE and CBPS approaches.

5.3 *Theoretical explanations*

The above empirical findings about the performance of the IPWEs with calibrated weights can be explained by the recent theoretical results in Tan (2020). Tan (2020) shows that under model misspecification, calibration with restrictions on covariate balance can reduce the relative error of the estimated weights compared to the true weights, which controls the mean squared errors of the IPWEs. The MLE approach for weight estimation focuses on reducing the absolute error of the estimated weights compared to the true weights, which is not directly connected to the mean squared errors of the IPWEs. The CBPS approach tries to maximize both the covariate balance (like the calibration approach) and prediction of treatment assignment (like the MLE approach). Therefore, its performance in terms of mean squared errors possibly lies in between the MLE and calibration approaches. Under model misspecification, improving covariate balance by the calibration and CBPS approaches can reduce the mean squared errors of the IPWEs and improve their efficiency. However, since empirical bias is quantified by averaging over repeated samples, depending on specific set-up

for model misspecification, the IPWE with weights from MLE can have similar or smaller bias than the IPWEs with weights from the calibration and CBPS approaches because large positive and negative differences from the true parameter values can be canceled out when averaging across samples.

In Web Appendix F, we also show why the MLE approach may perform poorly in terms of removing covariate imbalances asymptotically under mild model misspecification. Consequently, these covariate imbalances lead to poor performance of the corresponding IPWE even with large sample sizes.

6. Web Appendix F: Asymptotic covariate imbalances for the maximum likelihood approach under model misspecification

In this section, we show why the maximum likelihood approach can perform poorly in removing asymptotic imbalances of covariate distributions even under mild model misspecification. For clearer exposition, we focus on a point treatment, i.e., we drop the visit subscript. Following the weighting framework in Yiu and Su (2018), we can use the following measure to assess the covariate balance in the pseudo-population after weighting,

$$\frac{1}{n}\sum_{i=1}^{n} SW_i^A(\hat{\boldsymbol{\alpha}}, \hat{\boldsymbol{\beta}}) \frac{\partial}{\partial \boldsymbol{\beta}_{\mathrm{w}}} \log\{\mathrm{pr}(A_i \mid X_i; \boldsymbol{\beta}_{\mathrm{w}})\}\Big|_{\{\boldsymbol{\beta}_{\mathrm{wb}}=\hat{\boldsymbol{\alpha}}, \boldsymbol{\beta}_{\mathrm{wd}}=\mathbf{0}\}},$$

which is equal to the zero vector if and only if covariates are balanced in the current sample. Without loss of generality, let A_i and X_i be continuous, and $\boldsymbol{\alpha}^*$ and $\boldsymbol{\beta}^*$ be the probability limits of $\hat{\boldsymbol{\alpha}}$ and $\hat{\boldsymbol{\beta}}$, then this measure is asymptotically equivalent to

$$\begin{aligned}
&\mathrm{E}\left(SW_i^A(\boldsymbol{\alpha}^*, \boldsymbol{\beta}^*) \frac{\partial}{\partial \boldsymbol{\beta}_{\mathrm{w}}} \log\{\mathrm{pr}(A_i \mid X_i; \boldsymbol{\beta}_{\mathrm{w}})\}\Big|_{\{\boldsymbol{\beta}_{\mathrm{wb}}=\boldsymbol{\alpha}^*, \boldsymbol{\beta}_{\mathrm{wd}}=\mathbf{0}\}}\right) \\
&= \int_{X_i}\int_{A_i} \frac{\mathrm{pr}(A_i; \boldsymbol{\alpha}^*)}{\mathrm{pr}(A_i \mid X_i; \boldsymbol{\beta}^*)} \frac{\partial}{\partial \boldsymbol{\beta}_{\mathrm{w}}} \log\{\mathrm{pr}(A_i \mid X_i; \boldsymbol{\beta}_{\mathrm{w}})\}\Big|_{\{\boldsymbol{\beta}_{\mathrm{wb}}=\boldsymbol{\alpha}^*, \boldsymbol{\beta}_{\mathrm{wd}}=\mathbf{0}\}} \mathrm{pr}(A_i, X_i)\, dA_i\, dX_i \\
&= \int_{X_i}\int_{A_i} \frac{\mathrm{pr}(A_i \mid X_i)}{\mathrm{pr}(A_i \mid X_i; \boldsymbol{\beta}^*)} \frac{\partial}{\partial \boldsymbol{\beta}_{\mathrm{w}}} \mathrm{pr}(A_i \mid X_i; \boldsymbol{\beta}_{\mathrm{w}})\Big|_{\{\boldsymbol{\beta}_{\mathrm{wb}}=\boldsymbol{\alpha}^*, \boldsymbol{\beta}_{\mathrm{wd}}=\mathbf{0}\}} \mathrm{pr}(X_i)\, dA_i\, dX_i \\
&= \int_{X_i}\int_{A_i} \left(\frac{\mathrm{pr}(A_i \mid X_i)}{\mathrm{pr}(A_i \mid X_i; \boldsymbol{\beta}^*)} - 1\right) \frac{\partial}{\partial \boldsymbol{\beta}_{\mathrm{w}}} \mathrm{pr}(A_i \mid X_i; \boldsymbol{\beta}_{\mathrm{w}})\Big|_{\{\boldsymbol{\beta}_{\mathrm{wb}}=\boldsymbol{\alpha}^*, \boldsymbol{\beta}_{\mathrm{wd}}=\mathbf{0}\}} \mathrm{pr}(X_i)\, dA_i\, dX_i,
\end{aligned} \tag{2}$$

where the last line follows from the fact that under standard regularity assumptions, i.e., we can interchange differentiation and integration,

$$\int_{X_i}\int_{A_i} \frac{\partial}{\partial \boldsymbol{\beta}_{\mathrm{w}}} \mathrm{pr}(A_i \mid X_i; \boldsymbol{\beta}_{\mathrm{w}})\Big|_{\{\boldsymbol{\beta}_{\mathrm{wb}}=\boldsymbol{\alpha}^*, \boldsymbol{\beta}_{\mathrm{wd}}=\mathbf{0}\}} \mathrm{pr}(X_i)\ dA_i\ dX_i = \mathbf{0}.$$

From (2), it is clear that small asymptotic imbalances can be attained whenever the relative errors of the treatment probabilities are small, i.e., $|\mathrm{pr}(A_i \mid X_i)/\mathrm{pr}(A_i \mid X_i; \boldsymbol{\beta}^*) - 1| \approx 0$. Methods for estimating $\boldsymbol{\beta}$ that are targeted to achieving small absolute errors of the treatment probabilities, i.e., $|\mathrm{pr}(A_i \mid X_i) - \mathrm{pr}(A_i \mid X_i; \boldsymbol{\beta}^*)| \approx 0$, under mild model misspecification, such as least squares, are therefore not guaranteed to perform well in terms of reducing covariate imbalances. This is because, as mentioned in Section 5.3, small absolute errors do not necessarily imply small relative errors. Unfortunately, maximum likelihood for weight estimation is more geared towards achieving small absolute errors through minimizing the Kullback-Leibler divergence rather than small relative errors (Tan, 2020). As a result, the asymptotic covariate imbalances possibly from weights by maximum likelihood can lead to poor performance of the corresponding IPWE.

7. Web Appendix G: Further details of the HERS application

7.1 *Further details of the HERS cohort*

At the early period of the HERS from 1993 to 1995, treatment with a single ART was recommended for patients with CD4 cell counts less than 500. Beginning in late 1995 and early 1996, HAART (a combination of three or more ARTs) became more widely used in the HERS cohort. This increasing use of HAART is reflected in the right panel of Figure 2, which presents the crude percentages of patients who received HAART over the follow-up visits. Since the enrollment period of the HERS was between 1993 and 1995, the upward trend of HAART use started at visit 5, roughly two and half years into the study. The left panel of Figure 2 also presents the sample averages of the CD4 counts at each visit, which

show a decreasing trend up to visit 5. Then there is a level-off which coincides with the widespread use of HAART in this cohort. This phenomenon does not necessarily suggest the efficacy of HAART because it could be due to selection bias from dropout of patients with severe disease progression or other factors.

[Figure 2 about here.]

As mentioned in the main text, we are interested in quantifying the effect of HAART on the CD4 cell counts over time in the HERS. Because the HERS was an observational study, where therapies were not randomly assigned and varying over time, this leads to the potential for time-varying confounding between treatment and outcome. In particular, important HIV biomarkers such as CD4 cell counts and HIV viral load are affected by previous treatments, but also predict current treatment assignment and subsequent outcome measures over time. Moreover, estimation of the treatment effect may be further complicated by dependent censoring due to patient dropout, where about half of the 871 HIV-infected women at enrollment did not complete the study. More details can be seen in Table 5, which displays the number of patients who were HIV-positive at enrollment and still under follow-up, had their CD4 count observed, and dropped out at each visit.

[Table 5 about here.]

7.2 *Secondary sources of missing data in the HERS*

Besides attrition, there were secondary sources of missing data which resulted in intermittent missing data (before being lost to follow-up), missing data at enrollment for CD4 counts, and left-censored HIV viral load at the lower detection limit (LDL). We deal with these by following the approaches in Ko et al. (2003). Specifically, if there was one intermittent missing CD4 count value then the last observed value was carried forward; otherwise the patient was treated as having dropped out between their last observed visit and the next

visit. Missing CD4 values at enrollment were imputed from the patient's second visit, if possible; otherwise the patients missing CD4 values at visits 1 and 2 were excluded from the analysis. Finally, left-censored viral load values were imputed from a uniform distribution on the interval $[0, \mathrm{LDL}]$, where LDL was set at either 50 (13% of the viral load observations) or 500 (1% of the viral load observations) depending on the assays used.

7.3 *Covariates for the HERS analysis*

For the baseline covariates $\boldsymbol{V}_i$ in the MSMs fitted to the HERS data, we included the following variables at visit 7: $\log_{10}$ HIV viral load, HIV symptom level (5-point scale), status of one or two ARTs and status of HAART.

For the ordinal treatment model in the numerator of the stabilized inverse probability of treatment weights, we included the following covariates at visit j: visit indicators; status of one or two ARTs and status of HAART at visits $j-1$ and $j-2$. For the ordinal treatment model in the denominator, we additionally included: square root of CD4 count, $\log_{10}$ of HIV viral load and HIV symptom scale at visits $j-1$ and $j-2$; the two-way interactions between square root of CD4 count and status of HAART, square root of CD4 count and status of one or two ARTs, $\log_{10}$ of HIV viral load and status of HAART, $\log_{10}$ of HIV viral load and status of one or two ARTs, square root of CD4 count and $\log_{10}$ of HIV viral load at visit $j-1$; square root of CD4 count, $\log_{10}$ of HIV viral load and status of one or two ARTs at enrollment; site indicators; and race indicators (black, white, other).

7.4 *Estimated calibration functions and weights*

It is possible to gauge how well the weights from maximum likelihood adjust for confounding from observed covariates, by examining the standard deviation of the estimated calibration functions $c(\cdot, \hat{\boldsymbol{\lambda}})$ from the Type (1) method. A large non-zero value would provide evidence that substantial residual confounding still exists after weighting with weights from maximum likelihood. For IPTW, the mean and standard deviation of $c(\cdot, \hat{\boldsymbol{\lambda}})$ were 1.09 and 0.61,

which suggest that a fair amount of residual confounding from observed covariates has been addressed after applying the calibration to the weights from maximum likelihood.

Figure 3 displays boxplots of the estimated weights by visit in the HERS analysis. In all cases, it is clear that the estimated weights are not constant at any visit, which would suggest that treatment assignment and censoring depend on some of the covariates included in the models for these processes. Thus a naïve analysis, such as one without weighting, is unlikely to result in treatment effect estimates that are free of confounding and selection bias. However, as it is also clear that no weight exerts undue influence relative to the other weights, especially for IPTW only (where all weights are less than ten), there is no compelling evidence for non-positivity. Under the sequentially ignorability assumptions for treatment and censoring processes, this suggests that the parameters of the MSMs can be nonparametrically identified.

[Figure 3 about here.]

Figures 4 and 5 displays plots of the estimated calibration weights against their counterparts from maximum likelihood across visits for IPTW only and IPTCW. These figures show that the weights from the two approaches are not in complete correspondence, which suggests that there are chance imbalances of covariates in the treatment and censoring models after weighting by weights from maximum likelihood. Although the magnitude of chance imbalances does not appear to be too large (as most of the weights lie near the $y = x$ line), it is important to note that even small chance imbalances can lead to large estimation error in the IPWEs if the associations between these covariates and the outcome are strong (Imai et al., 2008).

[Figure 4 about here.]

[Figure 5 about here.]

7.5 *Short-term effects of HAART*

In addition to the marginal structural model specified in the main text, we also fit the following marginal structural model

$$\mathrm{E}(Y_{ij}^{\overline{a}_j}) = \delta_{0j} + \sum_{k=1}^{2} \delta_k I(D_i = k) + \boldsymbol{\delta}_v^\top \boldsymbol{V}_i + \sum_{k=0}^{2} I(D_i = k) \left\{ \gamma_{1k}(a_j^0 - a_j^1) + \gamma_{2k} a_j^1 \right\} \qquad (3)$$

for $j = 8, \ldots, 12$, where δ_{0j} are visit-specific intercept terms, $\boldsymbol{V}_i$ are baseline covariates evaluated at visit 7, and $\boldsymbol{\delta}_v$ are their corresponding regression coefficients, γ_{1k} and γ_{2k} represent strata-specific causal effects of recent exposures to one or two ARTs and HAART, respectively. This model quantifies the causal effect of receiving one or two ARTs and HAART in the previous six months on the current CD4 count, and therefore reflects the short-term treatment effects. Again, we also fit a marginal structural model without stratification by CD4 count at visit 7 in (3) .

Table 6 presents the results from fitting model (3) with weights estimated by the approaches described in the main text. Overall, these results exhibit a similar pattern to those reported in Table 1 of the main text, especially for the model without stratification. Applying inverse probability of treatment weighting with weights from maximum likelihood generally provides an upward adjustment to the estimated treatment effects when no weighting is applied, although with the cost of losing efficiency. In contrast, the calibration approach provides an even larger upward adjustment relative to the no weighting approach and is also more efficient. The consequences of adjusting for dependent censoring are again largely minor. Finally, the estimated effects of HAART are generally larger than those in Ko et al. (2003), except for the group CD4 counts > 500 at visit 7 where there is much uncertainty.

[Table 6 about here.]

While Ko et al. (2003) found therapeutic effects of HAART in this group with CD4 counts > 500, our analysis suggests that this could be a result of mixing the 'no HAART' group and the 'one or two ARTs' group, since we found that the effect of HAART only appears to be

therapeutic relative to one or two ARTs but not relative to no treatment when applying the maximum likelihood approach. In contrast, the calibration approach suggests that HAART is not even therapeutic relative to one or two ARTs in the group with CD4 counts > 500. However, since treatment history is not adjusted for in the MSM, there could be unmeasured confounding reflected in the treatment history that leads to these counter-intuitive results. Moreover, there is substantial uncertainty associated with the point estimates. Therefore, the evidence is not sufficient to draw a conclusion about the treatment effects for the baseline group with CD4 counts > 500.

References

Cao, W., Tsiatis, A. A., and Davidian, M. (2009). Improving efficiency and robustness of the doubly robust estimator for a population mean with incomplete data. *Biometrika* **96,** 723–734.

Chan, K. C. G., Yam, S. C. P., and Zhang, Z. (2016). Globally efficient nonparametric inference of average treatment effects by empirical balancing calibration weighting. *Journal of the Royal Statistical Society, Series B* **78,** 673–700.

Fong, C., Hazlett, C., and Imai, K. (2018). Covariate balancing propensity score for a continuous treatment: application to the efficacy of political advertisements. *Annals of Applied Statistics* **12,** 156–177.

Graham, B. S., Campos de Xavier Pinto, C., and Egel, D. (2012). Inverse probability tilting for moment condition models with missing data. *Rev. Econ. Stud.* **79,** 1053–1079.

Hainmueller, J. (2012). Entropy balancing for causal effects: multivariate reweighting method to produce balanced samples in observational studies. *Political Analysis* **20,** 24–46.

Han, P. (2016). Intrinsic efficiency and multiple robustness in longitudinal studies with drop-out. *Biometrika* **103,** 683–700.

Hernán, M. A., Brumback, B., and Robins, J. M. (2001). Marginal structural models to

estimate the joint causal effect of nonrandomized treatment. *J. Am. Stat. Assoc.* **96,** 440–448.

Imai, K., King, G., and Stuart, E. A. (2008). Misunderstandings between experimentalists and observationalists about causal inference. *Journal of the Royal Statistical Society: Series A (Statistics in Society)* **171,** 481–502.

Imai, K. and Ratkovic, M. (2014). Covariate balancing propensity score. *Journal of the Royal Statistical Society, Series B* **76,** 243–263.

Imai, K. and Ratkovic, M. (2015). Robust estimation of inverse probability weights for marginal structural models. *J. Am. Stat. Assoc.* **110,** 1013–1023.

Kallus, N. and Santacatterina, M. (2019). Optimal balancing of time-dependent confounders for marginal structural models. https://arxiv.org/abs/1806.01083v2.

Ko, H., Hogan, J. W., and Mayer, K. H. (2003). Estimating causal treatment effects from longitudinal hiv natural history studies using marginal structural models. *Biometrics* **59,** 152–162.

Robins, J., Sued, M., Lei-Gomez, Q., and Rotnitzky, A. (2007). Comment: performance of double-robust estimators when "inverse probability" weights are highly variable. *Statistical Science* **22,** 544–559.

Tan, Z. (2020). Regularized calibrated estimation of propensity scores with model misspecification and high-dimensional data. *Biometrika* **107,** 137–158.

Yiu, S. and Su, L. (2018). Covariate association eliminating weights: a unified weighting framework for causal effect estimation. *Biometrika* **105,** 709–722.

Zhou, X. and Wodtke, G. T. (2020). Residual balancing: A method of constructing weights for marginal structural models. *Political Analysis* pages 1–20. DOI: 10.1017/pan.2020.2.

Zubizarreta, J. R. (2015). Stable weights that balance covariates for estimation with incomplete outcome data. *J. Am. Stat. Assoc.* **110,** 910–922.

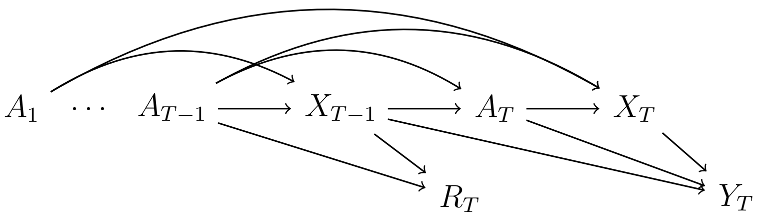

Figure 1. The relationship between the variables in the first simulation study.

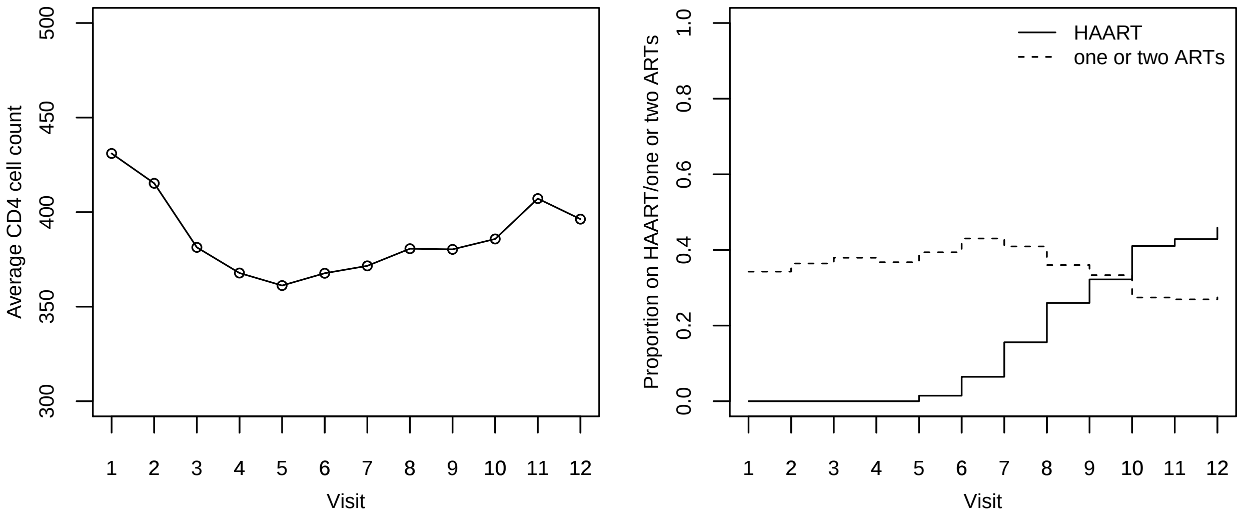

Figure 2. The average CD4 cell count and the proportion of patients who received HAART and one or two ARTs at each visit in the HERS data.

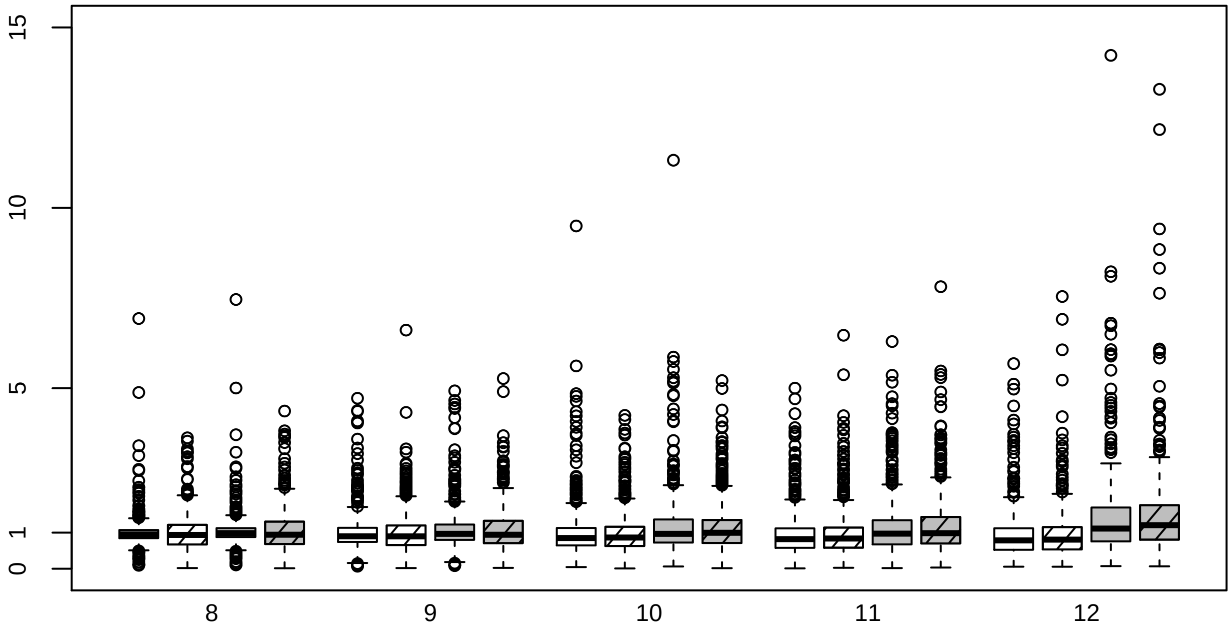

Figure 3. Box plots of the estimated weights stratified by visit in the HERS analysis in Section 7 of the main text. Left two white boxes: for IPTW only; right two gray boxes: for IPTCW; unshaded boxes: weights based on maximum likelihood; shaded boxes: calibrated weights by the Type (1) method.

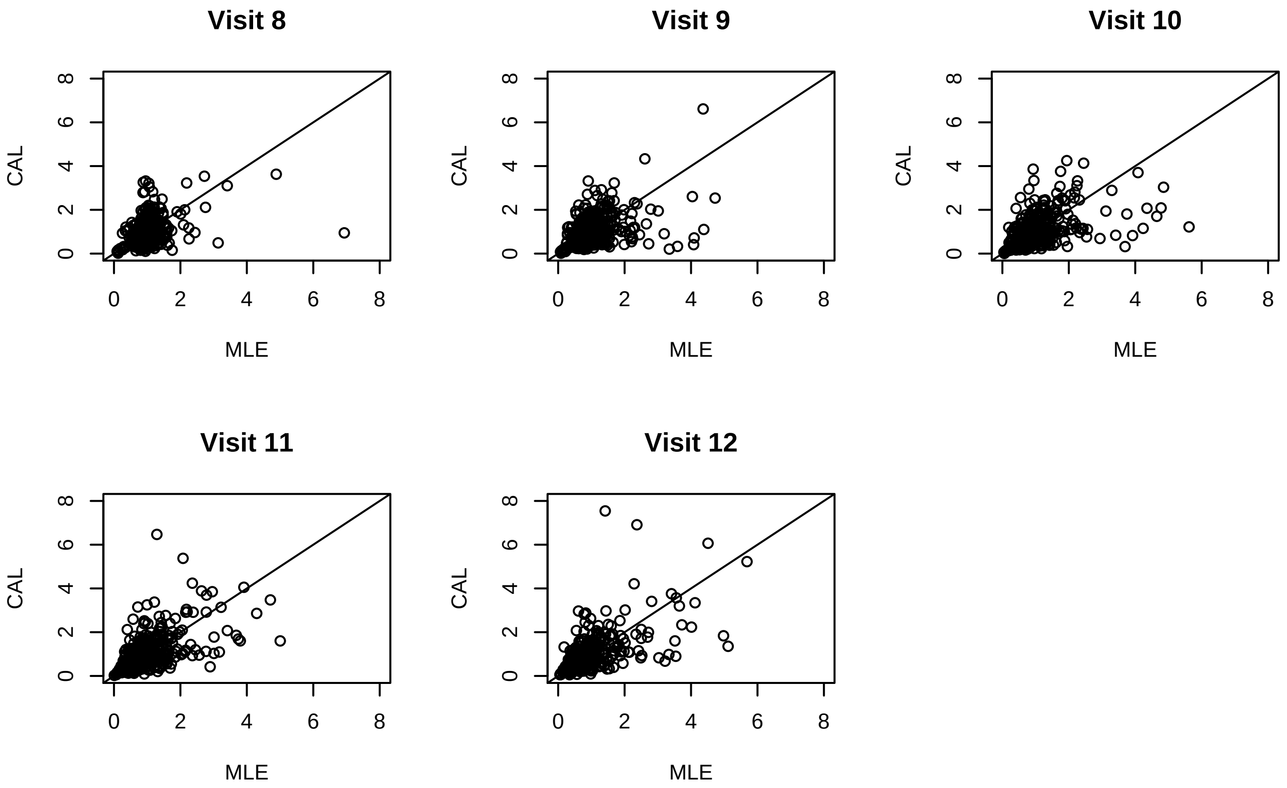

Figure 4. Plots of the estimated calibrated weights by the Type (1) method for IPTW only against their counterparts from maximum likelihood at each visit in the HERS.

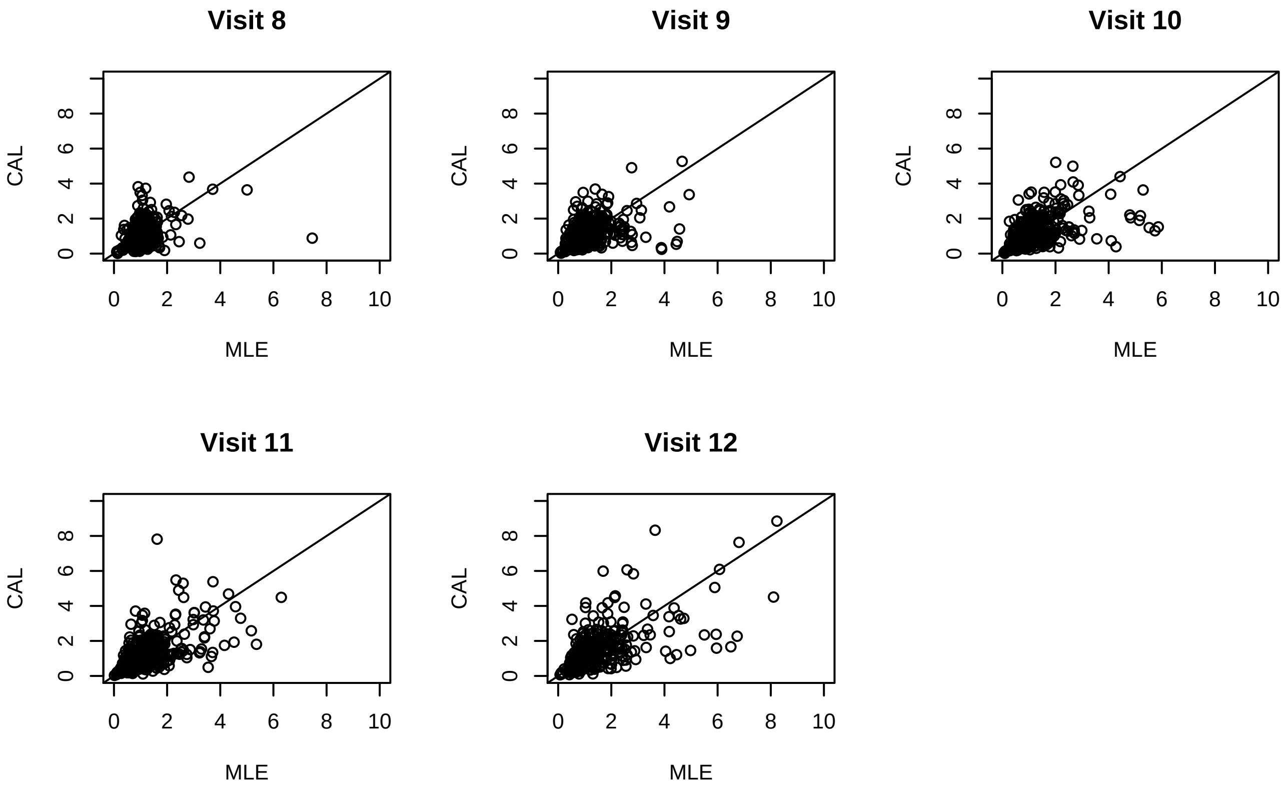

Figure 5. Plots of the estimated calibrated weights by the Type (1) method for IPTCW against their counterparts from maximum likelihood at each visit in the HERS.

Table 1
Data generating mechanism for the first simulation study

**Baseline** $(j = 0)$	
Censoring:	$R_0 = 1$
Treatment:	$A_0^0 \sim \text{Bernoulli}(0.5)$,
	$A_0^1 \mid A_0^0 = 1 \sim \text{Bernoulli}(0.5)$
Covariates:	$X_{01} = U_0 Z_{01}$,
	$X_{02} = U_0 Z_{02}$,
	$X_{03} = Z_{03} + 0.5(A_0^0 + A_0^1)$,
	$X_{04} = Z_{04} + 0.5(A_0^1 + A_0^1)$,
	where $U_0 = 1 - 0.3(A_0^0 + A_0^1)$, $\quad Z_{01}, Z_{02}, Z_{03}, Z_{04} \overset{i.i.d}{\sim} N(0,1)$
**Follow-up visits**	$(j = 1, \ldots, 10)$
Censoring:	$R_j \mid R_{j-1} = 1 \sim \text{Bernoulli}(q_j)$
	Scenario 1: $q_j = 1$ (no censoring)
	Scenario 2: $\text{logit}(q_j) = 1 + A_{j-1}^0 + A_{j-1}^1 + 0.5X_{j-1,1} + 0.5X_{j-1,2} + 0.2X_{j-1,3} + 0.2X_{j-1,4}$
Treatment:	$A_j^0 \sim \text{Bernoulli}(p_j)$,
	$A_j^1 \mid A_j^0 = 1 \sim \text{Bernoulli}(p_j)$
	$\text{logit}(p_j) = A_{j-1}^0 + A_{j-1}^1 + 0.5X_{j-1,1} + 0.5X_{j-1,2} - 0.2X_{j-1,3} - 0.2X_{j-1,4}$
Covariates:	$X_{j1} = U_j Z_{j1}$,
	$X_{j2} = U_j Z_{j2}$,
	$X_{j3} = Z_{j3} + 0.5 \sum_{t=0}^{j} (A_t^0 + A_t^1)$,
	$X_{j4} = Z_{j4} + 0.5 \sum_{t=0}^{j} (A_t^0 + A_t^1)$,
	where $U_j = 1 - 0.3(A_j^0 + A_j^1)$, $\quad Z_{j1}, Z_{j2}, Z_{j3}, Z_{j4} \overset{i.i.d}{\sim} N(0,1)$
Outcome:	$Y_j = 200 + 5(A_j^0 + A_j^1 + \sum_{t=j-1}^{j} \sum_{l=1}^{4} X_{tl}) + \epsilon_j,\ \epsilon_j \sim N(0, 20)$

Table 2
Data generating mechanism for the second simulation study

**Baseline** $(j = 0)$	
Treatment:	$A_0 \sim \text{Bernoulli}(0.5)$,
Covariates:	$X_{01} = U_0 Z_{01}$,
	$X_{02} = U_0 Z_{02}$,
	$X_{03} = Z_{03} + 0.5A_0$,
	$X_{04} = Z_{04} + 0.5A_0$,
	where $U_0 = 1 - 0.3A_0$, $\quad Z_{01}, Z_{02}, Z_{03}, Z_{04} \overset{i.i.d}{\sim} N(0, 1)$
**Follow-up visits**	$(j = 1, \ldots, 5)$
Treatment:	$A_j \sim \text{Bernoulli}(p_j)$,
	$\text{logit}(p_j) = A_{j-1} + 0.5X_{j-1,1} + 0.5X_{j-1,2} - 0.2X_{j-1,3} - 0.2X_{j-1,4}$
Covariates:	$X_{j1} = U_j Z_{j1}$,
	$X_{j2} = U_j Z_{j2}$,
	$X_{j3} = Z_{j3} + 0.5 \sum_{t=0}^{j} A_t$,
	$X_{j4} = Z_{j4} + 0.5 \sum_{t=0}^{j} A_t$,
	where $U_j = 1 - 0.3A_j$, $\quad Z_{j1}, Z_{j2}, Z_{j3}, Z_{j4} \overset{i.i.d}{\sim} N(0, 1)$
Outcome:	$Y_j = 200 + 5(A_j + \sum_{t=j-1}^{j} \sum_{l=1}^{4} X_{tl}) + \epsilon_j,\ \epsilon_j \sim N(0, 20)$

Table 3

Empirical bias, standard deviation (SD) and root mean squared error (RMSE) of the estimators of γ_1 and γ_2 in the marginal structural model from applying inverse probability of treatment weighting and inverse probability of treatment and censoring weighting to Scenarios 1 and 2, respectively, in the first simulation study. The weights are based on the maximum likelihood (MLE) and calibration (CMLE) approaches with Type (1) and Type (2) implementation methods.

	Scenario 1			Scenario 2		
	Bias (γ_1, γ_2)	SD (γ_1, γ_2)	RMSE (γ_1, γ_2)	Bias (γ_1, γ_2)	SD (γ_1, γ_2)	RMSE (γ_1, γ_2)
$n = 500$						
			correct covariates			
MLE	−0.00 0.03	0.67 0.66	0.67 0.66	−0.02 0.00	1.19 1.01	1.19 1.01
CMLE: Type (1)	−0.01 0.03	0.63 0.58	0.63 0.58	−0.03 −0.01	1.12 0.95	1.12 0.95
CMLE: Type (2)	−0.03 0.02	0.68 0.63	0.68 0.63			
			transformed covariates			
MLE	0.13 0.30	1.51 1.30	1.51 1.33	0.10 0.25	2.15 1.44	2.15 1.46
CMLE: Type (1)	0.14 0.27	0.53 0.44	0.55 0.51	0.07 0.17	0.94 0.77	0.95 0.79
CMLE: Type (2)	0.15 0.27	0.54 0.43	0.56 0.51			
$n = 1000$						
			correct covariates			
MLE	−0.01 0.03	0.51 0.57	0.51 0.57	−0.02 0.00	0.99 0.81	0.99 0.81
CMLE: Type (1)	−0.00 0.02	0.47 0.47	0.47 0.47	−0.02 −0.01	0.85 0.72	0.85 0.72
CMLE: Type (2)	−0.02 0.01	0.49 0.49	0.49 0.49			
			transformed covariates			
MLE	0.18 0.35	1.57 1.92	1.58 1.95	0.05 0.30	1.83 1.71	1.83 1.73
CMLE: Type (1)	0.14 0.29	0.40 0.34	0.43 0.45	0.09 0.17	0.75 0.61	0.76 0.63
CMLE: Type (2)	0.15 0.29	0.39 0.31	0.41 0.43			
$n = 2500$						
			correct covariates			
MLE	−0.01 0.00	0.34 0.46	0.34 0.46	0.02 0.00	0.69 0.63	0.69 0.63
CMLE: Type (1)	−0.01 0.01	0.31 0.36	0.31 0.36	0.01 −0.00	0.59 0.51	0.59 0.51
CMLE: Type (2)	−0.02 0.01	0.32 0.37	0.32 0.37			
			transformed covariates			
MLE	0.18 0.33	1.73 1.51	1.74 1.54	0.17 0.33	2.62 2.62	2.62 2.65
CMLE: Type (1)	0.14 0.29	0.32 0.27	0.36 0.39	0.07 0.15	0.57 0.47	0.57 0.49
CMLE: Type (2)	0.15 0.29	0.25 0.20	0.29 0.35			

Table 4

Empirical bias, standard deviation (SD), median absolute error (MAE), root mean squared error (RMSE) of the estimators for γ_1 in the marginal structural model from applying inverse probability of treatment weighting in the second simulation study. The weights are based on the maximum likelihood (MLE) approach, the calibration (CMLE) approach with Type (1) and Type (2) implementation methods and the CBPS approach.

	Correct covariates				*Transformed covariates*			
	MLE	CMLE Type (1)	CMLE Type (2)	CBPS	MLE	CMLE Type (1)	CMLE Type (2)	CBPS
$n = 500$								
Bias	-0.01	-0.01	-0.01	0.08	0.21	0.26	0.25	0.15
SD	0.47	0.47	0.47	0.85	1.09	0.45	0.43	0.86
MAE	0.32	0.31	0.31	0.57	0.40	0.35	0.34	0.58
RMSE	0.47	0.47	0.47	0.86	1.11	0.51	0.50	0.87
$n = 1000$								
Bias	-0.01	-0.01	-0.01	0.10	0.18	0.26	0.27	0.17
SD	0.33	0.33	0.33	0.62	1.48	0.33	0.30	0.67
MAE	0.22	0.22	0.22	0.43	0.35	0.30	0.29	0.46
RMSE	0.33	0.33	0.33	0.63	1.49	0.42	0.40	0.69
$n = 2500$								
Bias	0.01	0.01	0.01	0.11	0.13	0.28	0.28	0.18
SD	0.22	0.21	0.21	0.38	1.69	0.23	0.20	0.48
MAE	0.15	0.15	0.15	0.27	0.32	0.29	0.28	0.33
RMSE	0.22	0.21	0.21	0.40	1.69	0.36	0.34	0.51

Table 5
The number of patients who were HIV-positive at enrollment and still under follow-up, had their CD4 count observed, and dropped out at each visit in the HERS data.

Visit	1	2	3	4	5	6	7	8	9	10	11	12
In follow-up	871	815	787	748	702	668	636	614	595	571	553	449
CD4 observed	850	706	692	665	617	587	576	547	522	506	492	405
Dropped out	56	28	39	46	34	32	22	19	24	18	104	133

Table 6

Parameter estimates and their standard errors of the MSMs in (3) by applying no weighting, inverse probability of treatment weighting and inverse probability of treatment and censoring weighting with weights from maximum likelihood (MLE) and from the calibration approach (CMLE) by the Type (1) method to the HERS data.

Weight Estimation	Cumulative Effect	Strata by CD4 cell count at visit 7 < 200	200-500	> 500	No stratification
		No Weighting			
	$\leqslant$ 2 ARTs	23.67 (24.39)	45.60 (18.22)	-96.08 (43.26)	-0.88 (16.78)
	HAART	67.37 (24.51)	75.86 (19.94)	-94.15 (46.32)	28.93 (18.27)
		Treatment only			
MLE					
	$\leqslant$ 2 ARTs	42.50 (24.86)	48.40 (20.89)	-62.00 (51.89)	18.42 (20.10)
	HAART	80.92 (25.98)	77.53 (25.21)	-33.96 (59.05)	48.85 (23.24)
CMLE: Type (1)					
	$\leqslant$ 2 ARTs	25.92 (22.66)	69.53 (20.36)	-22.36 (42.27)	35.91 (16.37)
	HAART	81.64 (21.79)	87.17 (19.65)	-38.40 (46.75)	59.07 (16.32)
		Treatment and dropout			
MLE					
	$\leqslant$ 2 ARTs	38.67 (25.41)	47.90 (21.29)	-67.29 (51.72)	17.36 (19.92)
	HAART	79.47 (26.56)	77.27 (26.35)	-36.25 (58.11)	49.25 (23.27)
CMLE: Type (1)					
	$\leqslant$ 2 ARTs	25.39 (23.13)	71.23 (21.29)	-22.00 (42.40)	35.64 (16.82)
	HAART	82.15 (22.75)	83.95 (20.72)	-27.34 (44.32)	58.71 (16.85)
